# Distinct attentional characteristics of neurons with visual feature coding in the primate brain

**DOI:** 10.1126/sciadv.adq0332

**Published:** 2025-03-21

**Authors:** Jie Zhang, Runnan Cao, Xiaocang Zhu, Huihui Zhou, Shuo Wang

**Affiliations:** ^1^ Department of Radiology, Washington University in St. Louis, St. Louis, MO 63110, USA.; ^2^ Peng Cheng Laboratory, Shenzhen 518000, China.; ^3^ Shenzhen Institute of Advanced Technology, Chinese Academy of Sciences, Shenzhen 518055, China.

## Abstract

Visual attention and object recognition are two critical cognitive functions that shape our perception of the world. While these neural processes converge in the temporal cortex, the nature of their interactions remains largely unclear. Here, we systematically investigated the interplay between visual attention and stimulus feature coding by training macaques to perform a free-gaze visual search task with natural stimuli. Recording from a large number of units across multiple brain areas, we found that units exhibiting visual feature coding showed stronger attentional modulation of responses and spike–local field potential coherence than units without feature coding. Across brain areas, attention directed toward search targets enhanced the neuronal pattern separation of stimuli, with this enhancement more pronounced for units encoding visual features. Together, our results suggest a complex interplay between visual feature and attention coding in the primate brain, likely driven by interactions between brain areas engaged in these processes.

## Teaser

There is a complex interplay between visual feature and attention coding in the primate brain.

## INTRODUCTION

Visual attention and visual object recognition are integral processes that govern how animals make sense of their visual environment. The primate brain efficiently processes and interprets the vast array of visual information encountered in their surroundings. Visual attention serves as the gateway to this process by enhancing the perception of relevant information while filtering out distractions, allowing the brain to selectively prioritize specific stimuli for further processing (*1*–*3*). A substantial body of literature has documented the neural networks, pathways, and dynamics that dictate the brain’s selection, processing, and integration of information to achieve specific objectives (*4*, *5*). The complexity of visual attention involves a coordinated interaction among different brain regions and circuits, notably the prefrontal, parietal, and temporal cortices (*6*–*8*). These regions collaborate, playing a collective role in coordinating attentional resources and establishing intricate networks that dynamically adjust sensory processing in response to the goals and intentions of the animal (*9*). In particular, neurons in the inferotemporal (IT) cortex have been found to be modulated by attention, showing changes in neuronal responses (*10*–*13*), shifts in synchrony and coherence with other brain areas (*8*, *14*), and increased information for the exclusive representation of the attended object (*15*).

In conjunction with visual attention, visual object recognition—the cognitive process responsible for identifying and categorizing objects in the visual field—involves integrating various visual features, such as shape, color, and texture, to create a coherent representation of an object. This intricate process relies on a sophisticated interplay of neural circuits, enabling the brain to identify and categorize objects based on a myriad of visual features (*16*). IT neurons play a critical role in the representation and analysis of visual objects (*16*–*18*). In particular, IT neurons exhibit feature-based coding of objects, representing them across a broad and distributed population of neurons (*19*–*22*). In a particular form of feature-based coding known as axis-based feature coding, IT neurons parametrically correlate with visual features along specific axes in feature space (*23*–*26*). Neurons in downstream areas, such as the amygdala and hippocampus, likely receive this highly processed visual information as input and form high-level visual interpretations of stimuli (*27*). Furthermore, IT neurons exhibit visually selective responses to natural stimuli even when they are embedded in complex natural scenes (*10*).

The interaction between visual attention and object processing is a dynamic and intricate dance within the brain. Specifically, visual attention and feature coding converge on the IT and V4 regions. The parallel processing of stimulus features guides visual search (*28*), and, indeed, feature attention predicts the efficiency of target detection (*29*). While the analysis and encoding of visual features in V4 and IT neurons play a critical role in visual attention (*8*, *28*–*32*), the precise nature of the interaction between visual attention and neural object coding remains unclear. This study systematically addressed this question by training macaques to perform a free-gaze visual search task using natural face and object stimuli, allowing for detailed analysis of visual features. We simultaneously recorded a large number of units with a focal foveal receptive field (RF) across multiple attention and visual coding brain areas. We hypothesized that encoding of stimulus visual features and object-based attentional modulation converge in the temporal cortex, suggesting an integration of these two processes. We investigated whether neurons encoding visual features exhibited different attentional modulation of responses and spike–local field potential (LFP) coherence compared to those not encoding visual features. Our investigation also systematically examined the modulation of attention on neuronal representational geometry and whether such modulation was more pronounced for neurons encoding visual features.

## RESULTS

### Visual feature coding

**Fig. 1.**
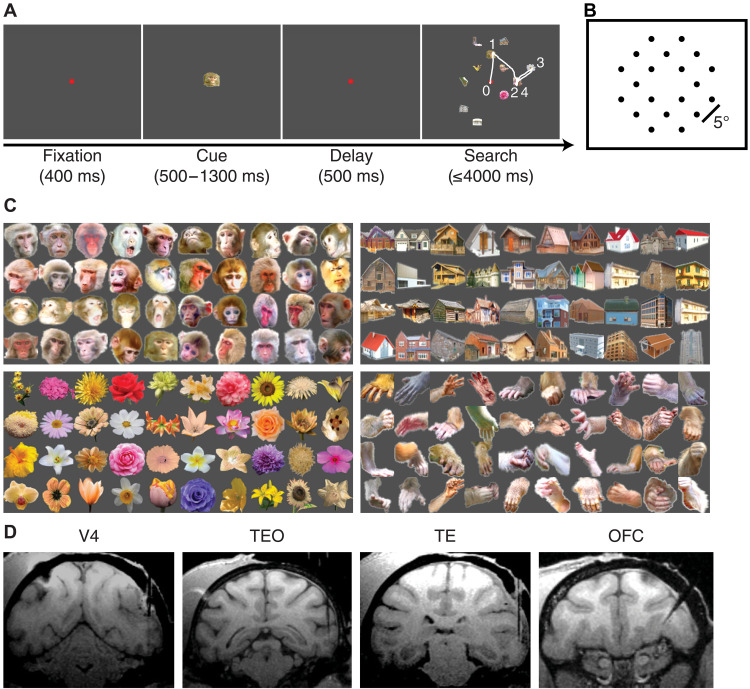
Task, stimuli, and recording sites. (**A**) Task. Monkeys initiated the trial by fixating on a central point for 400 ms. A cue was then presented for 500 to 1300 ms. After a delay of 500 ms, the search array with 11 items appeared. Monkeys were required to fixate on one of the two search targets that belonged to the same category as the cue for at least 800 ms to receive a juice reward. The white trace indicates eye gazes. The numbers indicate the order of the fixations. In this example, the monkey started from the center, fixated on one face target for less than 800 ms, moved to another face target and fixated for less than 800 ms, then moved to a distractor, and lastly shifted back to the face target, fixating for 800 ms to complete the trial. Note that this trial also included a return fixation (i.e., fixation no. 4) (*35*). (**B**) The 20 possible stimulus locations in the visual search task and the visually guided saccade task. Peripheral RFs were mapped using the visually guided saccade task, which had the same 20 possible stimulus locations as the visual search task. (**C**) Stimuli. Four categories of visual stimuli (40 images per category) were used for neural recordings. (**D**) Magnetic resonance imaging (MRI) images show the typical recording regions of V4, TEO, TE, and the orbitofrontal cortex (OFC).

Two monkeys performed a free-gaze visual search task with mapped RFs (see Materials and Methods and the summary of the number of units below), where their objective was to fixate on one of the two search targets that matched the category of the cue (Fig. 1A). Specifically, the monkeys were presented with a central fixation point for 400 ms, followed by a cue lasting 500 to 1300 ms. After a 500-ms delay, a search array appeared with 11 items, including two targets, randomly chosen from 20 possible locations (Fig. 1B). The monkeys had 4000 ms to find one target and maintain fixation on it for 800 ms to earn a juice reward. Fixating on either target completed the trial, and the monkeys did not search for the second target. A new trial began after the reward. The two target stimuli matched the category of the cue but were different images. In addition, the monkeys were required to maintain fixation throughout the cue and delay periods.

Both monkeys performed the task proficiently, with accuracy rates of 91.78 ± 0.19% for monkey S and 85.23 ± 0.41% for monkey E [see (*33*, *34*) for detailed behavioral and eye movement analyses]. The mean reaction time, from the onset of the search array to the onset of the last fixation, was 411.47 ± 67.01 ms (mean ± SD across sessions), and the mean fixation duration was 208.24 ± 153.77 ms (mean ± SD across fixations). On the basis of these behaviors, the monkeys could not adopt a strategy of serially sampling each search item for 800 ms without needing to remember the cue or attend to the cue category. However, the monkeys were allowed to implement any search strategy and sequence of fixations, and their unconstrained search behavior allowed for natural performance. In addition, the monkeys could revisit each search distractor or target as long as they did not fixate on a target for 800 ms. In 13.46 ± 7.00% of correct trials, the monkeys found the target after a return fixation (*35*). The monkeys were trained to search for faces and houses that belonged to the same visual category as the cue but were different images, allowing us to investigate visual attention while using a diverse range of images for studying visual object processing (Fig. 1C; see fig. S1 for characterization of the stimuli).

We recorded a total of 6871 units from area V4, 6694 units from TEO, 1947 units from TE, 5622 units from the orbitofrontal cortex (OFC), and 9916 units from the lateral prefrontal cortex (LPFC) (Fig. 1D; see fig. S2 for detailed characterization of the recording sites). Of these, 5070 units from area V4, 3800 units from TEO, 1251 units from TE, 1470 units from the OFC, and 2997 units from the LPFC exhibited a significant visually evoked response (i.e., their response to the cue or search array was significantly greater than their response to the baseline; Wilcoxon rank-sum test: *P* < 0.05). Foveal and peripheral RFs were mapped using the visual search task and an additional visually guided saccade task (see Materials and Methods; see also fig. S3 for response consistency between tasks). Among the visually responsive units, 1624 units from V4 (32.03%), 761 units from TEO (20.03%), 658 units from TE (52.60%), 888 units from the OFC (60.41%), and 32 units from the LPFC (1.07%) had a focal foveal RF. In this study, we focused on these units with a focal foveal RF to facilitate comparisons between visual feature coding and attention coding (excluding the limited number of units from the LPFC). The remaining units had localized peripheral RFs, broad foveal RFs, or unlocalized peripheral RFs (see Materials and Methods) (*33*).

**Fig. 2.**
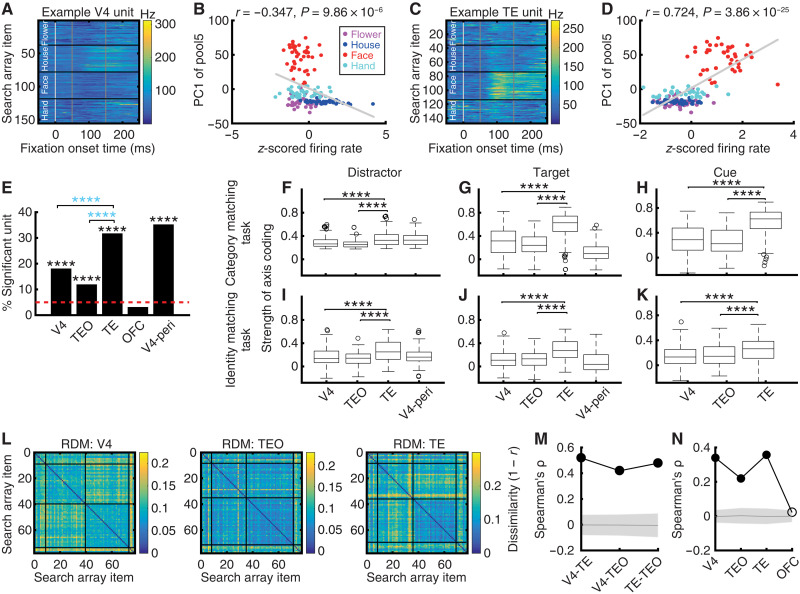
Axis-coding units. (**A** to **D**) Two example units showing axis-based feature coding. [(A) and (B)] V4. [(C) and (D)] TE. [(A) and (C)] Neural response to each search item. Color coding shows the firing rate (FR). The stimuli are sorted according to category. White vertical line: Fixation onset time. Gray vertical lines: The time window for visual feature coding analysis (50 to 150 ms after fixation onset). [(B) and (D)] Correlation between firing rate (*z*-scored) and PC1 of DNN features. Each dot represents a stimulus, and the gray line denotes the best fit from linear regression. Color coding indicates the object category. (**E**) The proportion of units demonstrating axis-based feature coding for each brain area. Black asterisks indicate a significant above-chance number of units at the population level. Blue asterisks indicate a significant difference between brain areas (χ^2^ test). ****P < 0.0001. (**F** to **K**) Strength of axis coding for each brain area. In each box plot, the central mark represents the median, box edges indicate the 25th and 75th percentiles, whiskers extend to nonoutlier extremes, and circles denote outliers. Asterisks indicate a significant difference between brain areas using a two-tailed two-sample *t* test. ****P < 0.0001. [(F) to (H)] Category-matching task. Monkeys were required to fixate on one of the two search targets that belonged to the same category as the cue. [(I) to (K)] Identity-matching task. There was only one search target, and monkeys were required to fixate on this identical search target as the cue. [(F) and (I)] Distractors. [(G) and (J)] Targets. [(H) and (K)] Cues. V4-peri: V4 units with a peripheral RF. (**L**) Representational dissimilarity matrices (RDMs) of search array items. Color coding shows dissimilarity values (1 − *r*). The stimuli are organized by category. (**M**) Correlation between neural RDMs. (**N**) Correlation between neural and DNN feature RDM. Solid: *P* < 0.05 (permutation test; Bonferroni-corrected across comparisons). Shaded area denotes ±SD across permutation runs.

Deep neural networks (DNNs) provide an unprecedented opportunity to effectively extract visual features from real-world natural images. These visual features are represented by the activations of DNN artificial units. Seminal studies have shown that the response of IT neurons can be explained by a linear combination of DNN features (*25*, *36*). In other words, the response of IT neurons can parametrically correlate with visual features along specific axes of the visual feature space, thus exhibiting an axis code for visual representation. Because the DNN features have a very high dimensionality (i.e., involving activations of a large number of DNN artificial units), partial least squares (PLS) regression is needed to fit the neural response (see fig. S4 for illustration). Here, we first analyzed visual feature coding in each brain area using established approaches that reveal whether a unit encodes a linear combination of DNN features (*25*, *36*) (see Materials and Methods for details). We used the mean firing rate in a time window of 50 to 150 ms after fixation onset as the response to each fixation (*37*, *38*). Specifically, using fixations on distractors that covered all stimulus items, units in V4, TEO, and TE exhibited an axis code for visual representation (see Fig. 2, A to D, for examples and Fig. 2E for group summary), consistent with prior studies showing axis coding in the primate IT and V4 (*23*–*26*). These units are referred to as axis-coding units. For illustration, the firing rates of example V4 (Fig. 2B) and TE (Fig. 2D) units were correlated with the first principal component (PC1) of the visual features, representing changes from inanimate (flowers and houses) to animate (faces) stimuli (see category labels in Fig. 2, B and D). TE (31.76%; binomial *P* = 2.20 × 10^−15^; Fig. 2E) had the highest percentage of axis-coding units, which was significantly higher than that of TEO (11.96%; binomial *P* = 4.31 × 10^−14^; χ^2^ test: *P* < 10^−20^) and V4 (18.10%; binomial *P* = 6.65 × 10^−37^; χ^2^ test: *P* = 9.99 × 10^−13^), whereas OFC did not have an above-chance number of axis-coding units (3.15%). We derived similar results when using DNN features from other layers (fig. S5, A to D), and our results also remained robust to features extracted using other DNNs (fig. S5, E and F).

The regression coefficients indicated the strength of axis coding (see Materials and Methods). We observed that TE exhibited the strongest axis coding compared to V4 and TEO (Fig. 2F; *P* < 0.0001), suggesting that the parametric encoding of complex visual features was most prominent in TE. Notably, axis-coding units, selected using fixations on distractors (Fig. 2E), also demonstrated a similar pattern of results during fixations on targets (Fig. 2G) and fixations on cues (Fig. 2H). In addition, we further replicated this finding using a separate identity matching task where monkeys were required to search for an identical target as the cue (in contrast to the above category matching task, there was only one search target in the identity matching task). Specifically, TE exhibited the strongest axis coding compared to V4 and TEO for fixations on distractors (Fig. 2I), fixations on targets (Fig. 2J), and fixations on cues (Fig. 2K; all *P* < 0.0001). All axis-coding units were selected during the category matching task using fixations on distractors (Fig. 2E; see also fig. S6); therefore, the strength of axis coding during the identity matching task (Fig. 2, I to K) represented a completely out-of-sample assessment. Furthermore, V4 units with a focal peripheral RF (i.e., only one search item was encompassed by the peripheral RF) had visual feature coding similar to that of foveal units (Fig. 2, F, G, I, and J; note that TEO and TE did not have sufficient units with a focal peripheral RF for this analysis).

We next analyzed whether a similar representation of visual features was shared across brain areas. Specifically, across distractor stimuli that were shared by the neural population of axis-coding units (note that fewer search array items were involved in Fig. 2L compared to all stimuli in Fig. 2, A and C), we used a representational similarity analysis (RSA) (*39*) (see Materials and Methods for details) to calculate the dissimilarity value (1 − Pearson’s *r*) in neural population response between each pair of stimuli (Fig. 2L). We then correlated the dissimilarity matrices (DMs) across brain regions to examine their representational similarity (Fig. 2M). The neuronal populations in V4, TEO, and TE (Fig. 2L) shared a similar representational structure (Fig. 2M; permutation *P* < 0.05, Bonferroni correction for multiple comparisons), suggesting that the neural representation of stimulus visual features was retained across brain areas. Similarly, we calculated DMs of DNN features between each pair of stimuli (i.e., pairwise distance in DNN features between stimuli using 1 − Pearson’s *r*) and then correlated the neural DMs (Fig. 2L) with the DNN DMs. The brain areas exhibiting axis coding (i.e., V4, TEO, and TE, but not OFC) also demonstrated representational similarity with the DNN feature space (Fig. 2N). Last, we correlated the strength of axis coding and the visual category selectivity index (SI) across units in each brain area (fig. S7; all units were included; axis-coding units exhibited an even stronger correlation; see fig. S7 legend for statistics) and confirmed that axis coding aligned with visual category selectivity.

Together, our results showed visual feature coding along the ventral processing pathway, with TE exhibiting the highest number of axis-coding units and the strongest axis coding. Moreover, axis coding of visual features was present in different attentional contexts (distractors versus targets versus cue) and tasks (category matching versus identity matching) and may be shared among brain areas.

### Axis-coding units exhibit a different attentional effect

**Fig. 3.**
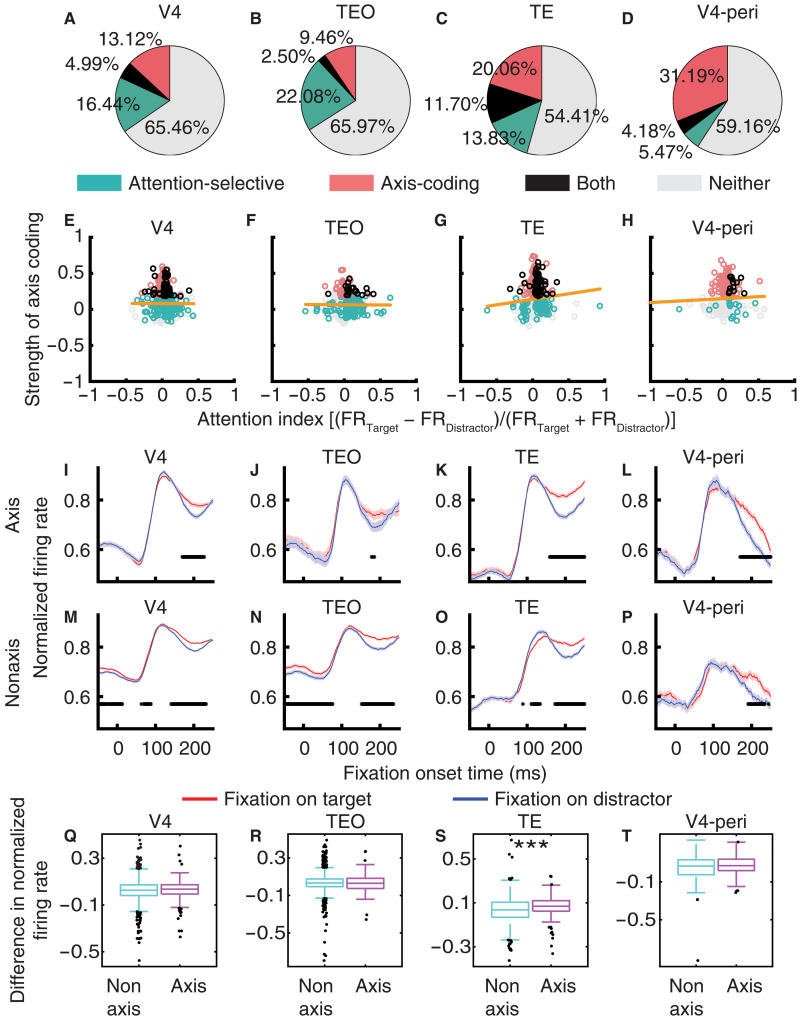
Attentional effect. (**A** to **D**) Population summary of attention-selective and axis-coding units. (**E** to **H**) Correlation between the strength of visual feature coding and the strength of attention coding [(FR_Target_ − FR_Distractor_)/(FR_Target_ + FR_Distractor_)]. Each circle represents a unit. Color coding shows the unit type (green: attention-selective; red: axis-coding; black: both; gray: neither). The yellow line is the linear fit for all units. (**I** to **P**) Attentional effect. [(I) to (L)] Axis-coding units. [(M) to (P)] Nonaxis-coding units. The firing rate of each unit was normalized to its maximum firing rate across conditions. Shaded area denotes ±SEM across units. The black bars illustrate the time points with a significant difference between fixations on targets (red) and fixations on distractors (blue; two-tailed paired *t* test, *P* < 0.05, Bonferroni-corrected across all time points). (**Q** to **T**) Comparison of attentional effect between axis-coding and nonaxis-coding units for each brain area. Each box plot shows the difference in normalized firing rate between fixations on targets and distractors (averaged from 150 to 225 ms after fixation onset). Asterisks indicate a significant difference using a two-tailed two-sample *t* test. ***P < 0.001. [(A), (E), (I), (M), and (Q)] V4. [(B), (F), (J), (N), and (R)] TEO. [(C), (G), (K), (O), and (S)] TE. [(D), (H), (L), (P), and (T)] V4 units with a focal peripheral RF.

How are axis-coding units related to attention coding? We first identified attention-selective units that differentiated fixations on targets and distractors (Materials and Methods). We used the mean firing rate in a time window of 150 to 225 ms after fixation onset as the response to each fixation. V4 (21.43%; binomial *P* = 6.65 × 10^−37^; Fig. 3A), TEO (24.57%; binomial *P* = 1.12 × 10^−17^; Fig. 3B), and TE (25.53%; binomial *P* = 2.20 × 10^−15^; Fig. 3C) all had an above-chance population of attention-selective units. Axis-coding units were more likely to be attention-selective units in TE [i.e., the proportion of attention-selective units within axis-coding units (*n*_Both_/*n*_Axis-Coding_) was significantly higher than the proportion of attention-selective units within all units (*n*_Attentive-Selective_/*n*_All_), χ^2^ test: *P* = 1.56 × 10^−3^, Bonferroni correction for comparisons in multiple brain areas; Fig. 3C], but this was not the case for V4 (Fig. 3A) or TEO (Fig. 3B). Therefore, these results indicate a significant relationship between visual feature coding and attention coding in TE but not in V4 or TEO. We further correlated the effects of visual feature coding and attention, for all units from a brain area regardless of selectivity. The strength of axis coding was weakly but significantly correlated with the attention index in TE (*r* = 0.085, *P* = 0.03; Fig. 3G) but not in V4 (Fig. 3E) or TEO (Fig. 3F).

We next investigated whether units with versus those without visual feature coding (i.e., axis coding) exhibited a differential response for attention. We first showed that across brain areas, both axis-coding (Fig. 3, I to K) and nonaxis-coding (Fig. 3, M to O) units exhibited attentional effects during visual search. However, axis-coding units exhibited a stronger attentional effect than nonaxis-coding units in TE [Fig. 3, K, O, and S; two-tailed two-sample *t* test: *t*(653) = 3.34, *P* = 9.01 × 10^−4^] but not in V4 [Fig. 3, I, M, and Q; *t*(1616) = 0.81, *P* = 0.42] or TEO [Fig. 3, J, N, and R; *t*(756) = 0.11, *P* = 0.91]. Therefore, attention coding was more integrated with visual feature coding in TE (see also fig. S8 for differential latency analyses). Furthermore, V4 units with a focal peripheral RF had a lower percentage of attention-selective units compared to those with a foveal RF (9.65%; binomial *P* = 6.18 × 10^−4^; Fig. 3D), and the attentional effect did not differ significantly between axis-coding and nonaxis-coding units [Fig. 3, L, P, and T; *t*(308) = 1.39, *P* = 0.17], as observed in V4 units with a foveal RF.

Together, we revealed units showing multiplexing functions for both attention and visual feature coding, with these two forms of coding primarily converged at TE. Units exhibiting visual feature coding had a stronger attentional effect in TE, suggesting an interaction between attention and visual feature coding.

### Attention modulates neural object representations

Above, we demonstrated a stronger response for axis-coding units when they encode visual attention. How does attention modulate neural object representations? Our previous work has shown that familiarity and familiarization modulate the population geometry of faces (*40*). Using this established approach, we quantified the population representational geometry of the units as a function of attentional contexts. If all units change their response proportionally, then the angle between the neuronal vectors will not change; otherwise, a change in the angle will suggest a change in the population geometry.

**Fig. 4.**
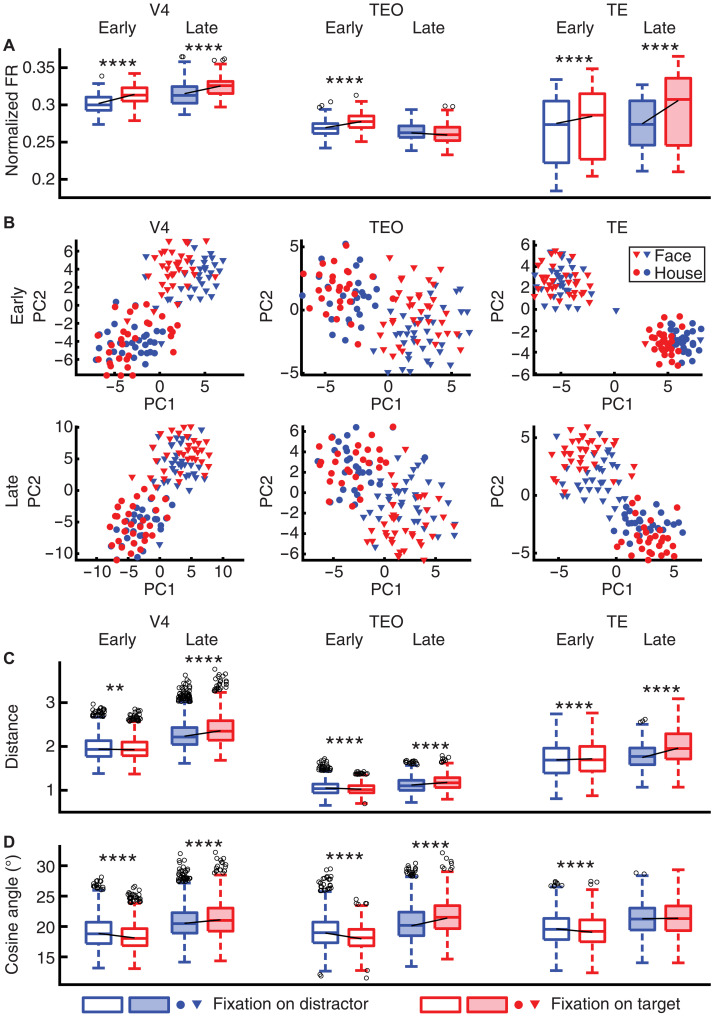
Attentional modulation of neuronal representational geometry. (**A**) Normalized firing rate. (**B**) Distribution of stimuli in the neuronal feature space (constructed using PCA of the average neural response). Dimensions of the neuronal feature space are represented by the PCs. Each triangle represents a face stimulus, and each dot represents a house stimulus. Early: 50 to 150 ms after fixation onset. Late: 150 to 225 ms after fixation onset. Red: Fixations on targets. Blue: Fixations on distractors. (**C**) Representational distance for the population of neurons. (**D**) Angle between the neuronal vectors. Open boxes: Early time window (50 to 150 ms after fixation onset). Solid boxes: Late time window (150 to 225 ms after fixation onset). Asterisks indicate a significant difference between conditions (distractor versus target) using a two-tailed paired *t* test. **P < 0.01, and ****P < 0.0001.

First, in axis-coding units, attention (i.e., fixations on targets compared to fixations on distractors) increased firing rate in the late time window (150 to 225 ms after fixation onset; Fig. 4A) in V4 [two-tailed paired *t* test: *t*(63) = 8.20, *P* = 1.57 × 10^−11^] and TE [*t*(61) = 5.97, *P* = 1.29 × 10^−7^]. Both the neuronal distance [Fig. 4C; see Fig. 4B for illustration; V4: *t*(2015) = 28.93, *P* = 3.34 × 10^−154^; TEO: *t*(2015) = 15.52, *P* = 2.16 × 10^−51^; TE: *t*(1890) = 35.46, *P* = 1.56 × 10^−211^] and the angle between the neuronal vectors [Fig. 4D; V4: *t*(2015) = 14.95, *P* = 5.15 × 10^−48^; TEO: *t*(2015) = 15.80, *P* = 3.92 × 10^−53^; except TE: *t*(1890) = 1.40, *P* = 0.16] increased for targets compared to distractors, suggesting that neural representations of targets became more distinct, which could, in turn, facilitate target detection. Notably, such enhancement in neuronal representational distance and angle even happened in TEO where firing rate did not increase for search targets [Fig. 4A; *t*(63) = −1.96, *P* = 0.054], suggesting that changes in neuronal representational geometry could be dissociated from changes in firing rate.

Second, in the early time window (50 to 150 ms after fixation onset), although firing rate increased for fixations on targets across brain areas [Fig. 4A; V4: *t*(63) = 11.77, *P* = 1.49 × 10^−17^; TEO: *t*(63) = 6.89, *P* = 3.05 × 10^−9^; TE: *t*(61) = 6.30, *P* = 3.58 × 10^−8^], attention did not enhance the neuronal distance [Fig. 4C; V4: *t*(2015) = −3.15, *P* = 0.0017; TEO: *t*(2015) = −7.73, *P* = 1.71 × 10^−14^; except TE: *t*(1890) = 6.26, *P* = 4.76 × 10^−10^] or the angle between the neuronal vectors [Fig. 4D; V4: *t*(2015) = −21.92, *P* = 1.14 × 10^−95^; TEO: *t*(2015) = −16.06, *P* = 1.12 × 10^−54^; TE: *t*(1890) = −7.48, *P* = 1.13 × 10^−13^], suggesting that attention enhanced pattern separation in a later stage.

**Fig. 5.**
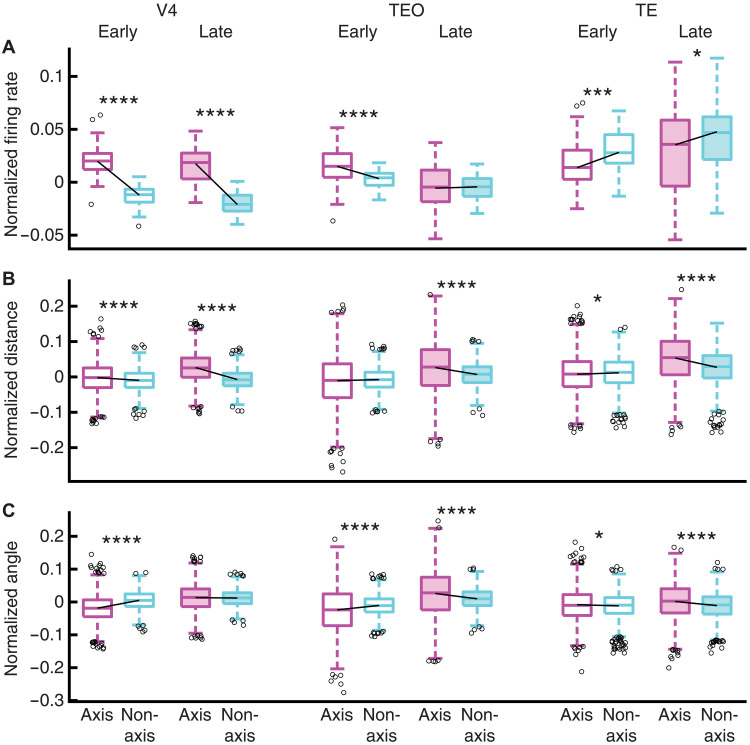
Differential attentional modulation of neuronal representational geometry between axis-coding and nonaxis-coding units. (**A**) Normalized firing rate [(FR_Target_ − FR_Distractor_)/(FR_Target_ + FR_Distractor_)]. (**B**) Normalized representational distance for the population of neurons [(Distance_Target_ − Distance_Distractor_)/(Distance_Target_ + Distance_Distractor_)]. (**C**) Normalized angle between the neuronal vectors [(Angle_Target_ − Angle_Distractor_)/(Angle_Target_ + Angle_Distractor_)]. Open boxes: Early time window (50 to 150 ms after fixation onset). Solid boxes: Late time window (150 to 225 ms after fixation onset). Asterisks indicate a significant difference between axis-coding and nonaxis-coding units using a two-tailed two-sample *t* test. *P < 0.05, ***P < 0.001, and ****P < 0.0001.

Third, we examined whether attentional modulation of neuronal representations was particularly pronounced for axis-coding units compared to nonaxis-coding units (Fig. 5). In the late time window, axis-coding units across brain areas exhibited a stronger attentional modulation for both the neuronal distance [Fig. 5B; V4: *t*(3667) = 29.07, *P* = 2.40 × 10^−167^; TEO: *t*(3725) = 10.16, *P* = 5.87 × 10^−24^; TE: *t*(3719) = 13.90, *P* = 7.69 × 10^−43^] and the angle between the neuronal vectors [Fig. 5C; TEO: *t*(3725) = 8.77, *P* = 2.77 × 10^−18^; TE: *t*(3719) = 7.77, *P* = 1.00 × 10^−14^; except V4: *t*(3667) = 1.04, *P* = 0.30], showing a disproportionate attentional modulation for units encoding visual features. Changes in neuronal representations were greater for axis-coding units even if changes in firing rate were greater [Fig. 5A; V4: *t*(120) = 14.79, *P* = 8.02 × 10^−29^], smaller [TE: *t*(121) = −2.12, *P* = 3.58 × 10^−2^], or similar [TEO: *t*(121) = 0.05, *P* = 0.96] compared to nonaxis-coding units, again indicating a dissociation between firing rate and neuronal representational geometry.

Last, in the early time window, although changes in firing rate were significantly different between axis-coding and nonaxis-coding units (Fig. 5A; all *P* < 0.001), we did not observe a consistent pattern of differences in neuronal representational geometry (Fig. 5, B and C). Together, attention to search targets enhanced pattern separation of the stimuli across brain areas, and such enhancement was more pronounced for units encoding visual features.

### Disproportionate target-induced desynchronization for axis-coding versus nonaxis-coding units

Are axis-coding and nonaxis-coding units part of the same functional network? To address this question, we analyzed the coherence between spikes and LFPs recorded simultaneously across brain areas within a time window of 0 to 200 ms relative to fixation onset during the search task (see Materials and Methods). We included spikes from V4 and IT (OFC and LPFC were excluded because of having fewer than 20 axis-coding units) and LFPs from all four brain areas. TE and TEO were combined into IT because they were not recorded simultaneously. However, separating TE and TEO in the analyses yielded similar results (fig. S9).

**Fig. 6.**
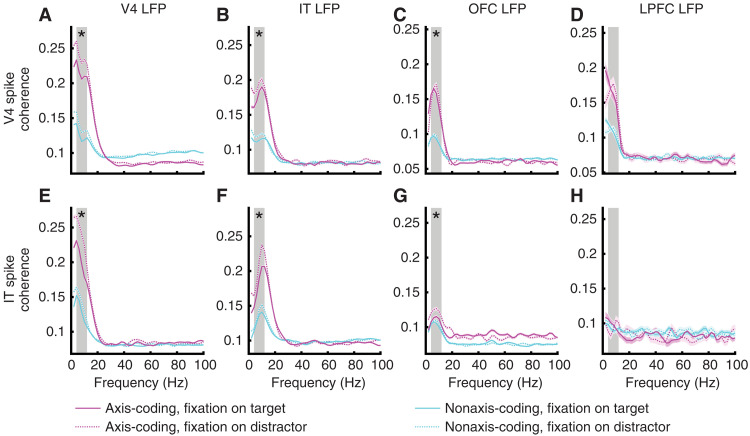
Spike-LFP coherence. (**A**) V4 spike–V4 LFP. (**B**) V4 spike–IT LFP. (**C**) V4 spike–OFC LFP. (**D**) V4 spike–LPFC LFP. (**E**) IT spike–V4 LFP. (**F**) IT spike–IT LFP. (**G**) IT spike–OFC LFP. (**H**) IT spike–LPFC LFP. Magenta: Axis-coding units. Cyan: Nonaxis-coding units. Solid line: Fixation on targets. Dotted line: Fixation on distractors. Magenta- and cyan-shaded areas denote ±SEM across spike-LFP pairs. Gray-shaded area denotes the theta frequency band (4 to 12 Hz). Asterisks indicate a significant difference in target-induced desynchronization (i.e., the reduction in spike-LFP coherence for fixations on targets compared to fixations on distractors, averaged across the theta frequency band) between axis-coding and nonaxis-coding units using a two-tailed two-sample *t* test. *P < 0.05.

First, axis-coding units exhibited significantly greater spike-LFP coherence compared to nonaxis-coding units across various brain regions (similar results were obtained using the same number of spike-LFP pairs): between V4 spike and V4 LFP [Fig. 6A; two-tailed two-sample *t* test: *t*(58810) = 112.81, *P* < 10^−50^], between V4 spike and IT LFP [Fig. 6B; *t*(15604) = 52.83, *P* < 10^−50^], between V4 spike and OFC LFP [Fig. 6C; *t*(9002) = 40.47, *P* < 10^−50^], between V4 spike and LPFC LFP [Fig. 6D; *t*(1380) = 11.67, *P* = 4.29 × 10^−30^], between IT spike and V4 LFP [Fig. 6E; *t*(15960) = 59.98, *P* < 10^−50^], between IT spike and IT LFP [Fig. 6F; *t*(45318) = 74.13, *P* < 10^−50^], and between IT spike and OFC LFP [Fig. 6G; *t*(10076) = 10.22, *P* < 10^−50^; but not between IT spike and LPFC LFP; Fig. 6H; *t*(488) = 0.20, *P* = 0.84]. These findings suggest that axis-coding units displayed generally stronger synchronization across brain areas. However, we derived similar results by subtracting the baseline spike-LFP coherence during the initial fixation preceding the cue (within a time window of 0 to 200 ms relative to the onset of the initial fixation; fig. S10).

For both axis-coding and nonaxis-coding units, spikes desynchronized with LFPs in the theta frequency band for fixations on targets compared to fixations on distractors (Fig. 6). Compared to nonaxis-coding units, axis-coding units demonstrated a stronger target-induced desynchronization between V4 spike and V4 LFP {Fig. 6A; two-tailed two-sample *t* test on normalized coherence [(Coherence_Distractor_ − Coherence_Target_)/(Coherence_Distractor_ + Coherence_Target_)]: *t*(29404) = 2.98, *P* = 0.00288}, between V4 spike and IT LFP [Fig. 6B; *t*(7801) = 2.06, *P* = 0.0395], between V4 spike and OFC LFP [Fig. 6C; *t*(4500) = 2.03, *P* = 0.0424], between IT spike and V4 LFP [Fig. 6E; *t*(7979) = 10.27, *P* = 1.42 × 10^−24^], between IT spike and IT LFP [Fig. 6F; *t*(22658) = 9.36, *P* = 8.77 × 10^−21^], and between IT spike and OFC LFP [Fig. 6G; *t*(5037) = 4.62, *P* = 4.01 × 10^−6^], suggesting that axis-coding units disproportionately engaged the attention network compared to nonaxis-coding units. The systematic differences between axis-coding and nonaxis-coding units also indicated differential top-down modulation from the PFC. Last, we confirmed that our results could not be attributed to superior data quality or spike sorting quality for axis-coding units compared to nonaxis-coding units (figs. S11 to S13).

### Directional theta influence across brain areas for axis-coding versus nonaxis-coding units

**Fig. 7.**
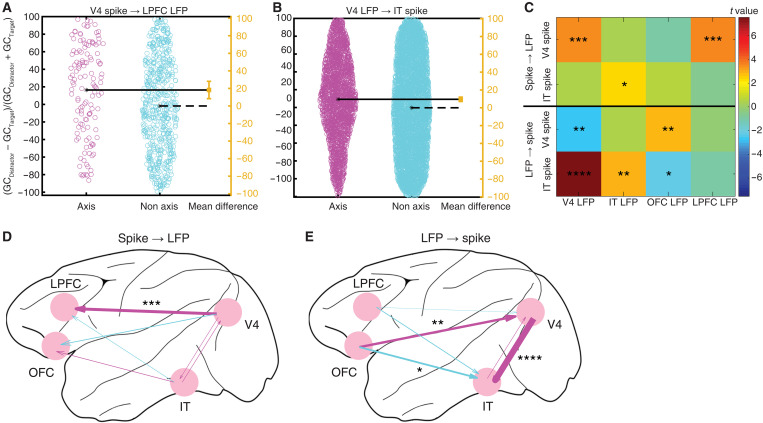
Granger causality. (**A** and **B**) Compared to nonaxis-coding units, axis-coding units had a stronger reduction in Granger causality (GC) between brain areas [(GC_Distractor_ − GC_Target_)/(GC_Distractor_ + GC_Target_)]. Each circle represents a spike-LFP pair. The mean of the nonaxis-coding units corresponds to the zero effect size, and the mean of the axis-coding units corresponds to the value of the effect size on the effect size axis (yellow). The vertical error bar in yellow indicates the actual mean-difference effect size value and the confidence intervals. (A) V4 spike influence on LPFC LFP. (B) V4 LFP influence on IT spike. (**C**) Summary of Granger causality for each spike-LFP pair. Color coding shows the *t* values of the two-tailed two-sample *t* test (axis − nonaxis). Asterisks indicate a significant difference between axis-coding and nonaxis-coding units using a two-tailed two-sample *t* test. *P < 0.05, **P < 0.01, ***P < 0.001, and ****P < 0.0001. (**D** and **E**) Differences in target-induced reduction of cross-area Granger causality between axis-coding and nonaxis-coding units. The thickness of the arrow is proportional to the *t* value of the two-tailed two-sample *t* test. Red: Axis-coding > nonaxis-coding. Blue: Nonaxis-coding > axis-coding. (D) Spike influence on LFP. (E) LFP influence on spike.

To investigate the direction of interactions between brain areas, we performed a Granger causality analysis based on spikes and LFPs in the theta frequency band (see fig. S14 for analyses across frequencies). We first analyzed the influence of spikes on LFPs (Fig. 7, A, C, and D, and fig. S14, A to H). Attention modulated interactions between brain areas when comparing fixations on targets versus distractors, with targets inducing a decrease in Granger causality (i.e., desynchronization). Such modulation was more pronounced for axis-coding than nonaxis-coding units (Fig. 7, C and D). Specifically, the influence of V4 spike on V4 LFP [fig. S14A; two-tailed two-sample *t* test: *t*(29404) = 3.54, *P* = 4.01 × 10^−4^], the influence of V4 spike on LPFC LFP [Fig. 7A and fig. S14D; *t*(689) = 3.62, *P* = 3.19 × 10^−4^], and the influence of IT spike on IT LFP [fig. S14F; *t*(22658) = 2.46, *P* = 0.0138] were more strongly modulated in axis-coding units compared to nonaxis-coding units.

We also analyzed the influence of LFPs on spikes (Fig. 7, C and E, and fig. S14, I to P). Again, attention modulated interactions between brain areas, and such modulation was disproportionate for axis-coding units (Fig. 7, C and E). Notably, the influence of OFC LFP on V4 spike [fig. S14K; *t*(4500) = 2.91, *P* = 0.00367], the influence of V4 LFP on IT spike [Fig. 7B and fig. S14M; *t*(7979) = 7.54, *P* = 5.05 × 10^−14^], and the influence of IT LFP on IT spike [fig. S14N; *t*(22,658) = 2.98, *P* = 0.00292] were more strongly modulated in axis-coding units, whereas the influence of V4 LFP on V4 spike [fig. S14I; *t*(29,404) = 2.60, *P* = 0.00930] and the influence of OFC LFP on IT spike [fig. S14O; *t*(5037) = 2.19, *P* = 0.0285] were more strongly modulated in nonaxis-coding units.

Together, our results reveal bidirectional influences between spikes and LFPs across brain areas. In particular, attentional modulation of these influences is more pronounced for axis-coding units than nonaxis-coding units, suggesting that axis-coding units differentially engage the attention neural network.

## DISCUSSION

In this study, we recorded from a large number of units across attention and visual coding areas. TE units exhibiting visual feature coding (i.e., axis-coding units) showed stronger attentional modulation of responses compared to nonaxis-coding units, indicating an integration of attention and visual feature coding in these units. Across brain areas, attention to search targets enhanced neuronal pattern separation of the stimuli, and such enhancement was more pronounced for axis-coding units. Moreover, axis-coding units exhibited greater target-induced spike-LFP desynchronization to other brain areas when encoding attention, suggesting differential engagement with the attention neural network. Our results indicated two stages of processing. In the early stage (50 to 150 ms after fixation onset), neurons primarily processed visual features and exhibited axis coding. In the late stage (150 to 225 ms after fixation onset), neurons primarily implemented attentional modulation of neuronal responses. Together, our results suggest an intricate interplay between visual feature coding and attention coding in the primate brain, which can be attributed to the differential interactions between brain areas engaged in these processes.

We trained macaques to perform a free-gaze visual search task using natural face and object stimuli, enabling detailed analysis of visual features. Our study is thus uniquely positioned to investigate the intricate interplay between attention coding and visual feature coding. We identified units encoding both visual features and attention, resembling the multidimensional processing observed in the primate amygdala, where the same neurons encode valence, arousal, and visual features (*41*). Neurons encoding visual features may form the basis of feature-based attention. Consistent with our present finding, it has been shown using a similar naturalistic free-gaze visual search task that V4 neurons exhibit not only visually driven response to features but also top-down modulation of response to search targets (*31*). Notably, we found that units encoding visual features exhibited a different attentional effect and spike-LFP coherence. While we focused on the more recent axis-coding model in the present study, our previous research also demonstrated that units with visual category selectivity exhibit stronger attentional modulation of responses (*42*), suggesting an interaction between visual category selectivity and attention. Similar to our present findings, units in the human amygdala and hippocampus not only encode a visual attentional effect toward search targets but also encode visual categories (*43*); however, in contrast, the units encoding attention and visual categories appear to be independent. This raises the question of whether and where these two processes diverge along the processing stream, a question that future analyses should address.

While feature-selective responses in the macaque visual cortex adapt to eye movements during natural vision (*44*), the location-independent nature of feature-based attention is especially effective for selectively modulating the neural representations of stimuli or specific elements within complex visual scenes that align with the feature currently being attended to (*45*). In this study, attention modulated neural representational geometry, supporting a recent hypothesis that attention improves performance by reshaping stimulus representations to align with the readout (*46*). Our present results can be interpreted in the framework of pattern separation (*47*, *48*), the process of transforming similar representations or memories into dissimilar, nonoverlapping representations, and are in line with the tuning sharpening in the primate IT cortex (*15*, *49*, *50*). The representational geometry changed differently in axis-coding versus nonaxis-coding units, and we revealed the temporal dynamics (and specificity) for visual feature coding and attentional modulation. Furthermore, while neuronal representational distance and angle were calculated on the basis of firing rate, changes in these measures could be dissociated from changes in firing rate (Figs. 4 and 5).

We observed a desynchronization for attended stimuli (fixations on search targets) in the theta frequency band, as opposed to the same stimuli when they were unattended (fixations on distractors) in both V4 and IT. This finding aligns with previous research demonstrating desynchronization for attended stimuli in V4 within a similar frequency band (*51*, *52*). Desynchronization has been observed in instances of feature-based attention, where discrimination between target and distractor in the peripheral RF occurs, as well as during saccade selection, involving directing attention into (attention in) or out of (attention out) the peripheral RF. This pattern was evident in V4 spike–V4 LFP coherence, V4 spike–frontal eye field LFP coherence, and frontal eye field spike–V4 LFP coherence (*52*). Furthermore, spike-field Granger causality can be used to reveal the modulatory effects that are inaccessible by traditional methods, such that spike→LFP Granger causality is modulated by the behavioral task, whereas LFP→spike Granger causality is mainly related to the average synaptic input (*53*). Notably, in this study, we observed bidirectional differences in Granger causality between axis-coding and nonaxis-coding units.

Our present result is consistent with top-down modulation from the frontal cortex to the temporal lobe (*43*, *54*). It has been shown that attention to faces versus houses enhances the sensory responses in the fusiform face area (FFA) and parahippocampal place area (PPA), respectively. The increases in sensory responses are accompanied by induced gamma synchrony between the inferior frontal junction (IFJ) and either the FFA or PPA, depending on which object is attended, and the IFJ directs the flow of visual processing during object-based attention, at least, in part, through coupled oscillations with specialized areas such as the FFA and PPA (*55*). In addition, individual PFC units synchronize to the LFP ensemble corresponding to the current task goal or rule (*56*), consistent with our prior report showing that different search goals (social versus nonsocial) differentially engage the PFC (*34*). Our present result is also consistent with the rhythmic theory of attention that both perceptual sensitivity during covert spatial attention and the probability of overt exploratory movements are tethered to theta-band activity in the attention network (*57*).

Our current results can be linked to computational models and theories of visual search. First, our findings provide neural evidence that aligns with key components of the Guided Search 6.0 (GS6) computational model (*58*). Specifically, we observed that neurons in the temporal cortex exhibit distinct attentional modulation of responses depending on their feature-coding properties, supporting the GS6 model’s concept of selective attention modulating neural activity based on visual feature processing. This is consistent with the model’s idea of a priority map, where attention is guided by both top-down and bottom-up feature guidance. In particular, our results suggest that the early stage of neural processing, which we identified as primarily focused on visual feature coding, may correspond to GS6’s preattentive feature extraction phase, in which visual features are processed in parallel to form a priority map that guides attention. The later stage of processing, where attention modulates neural responses more strongly, aligns with the model’s focus on selective attention and the recognition of targets or rejection of distractors. Second, an inverse reinforcement learning (IRL) model predicts human gaze behavior and internal belief states during goal-directed search (*59*). In our study, feature-coding neurons were more engaged during attentional processing, aligning with the IRL model’s concept of learned prioritization of certain visual features to guide eye movements. Last, our findings on visual feature coding, combined with our previous detailed analyses of face-specific attention (*34*) and visual fields (*33*) during visual search, may suggest neural underpinnings for inherent biases in attention when perceiving natural scenes and eccentricity-dependent recognition, which can, in turn, help explain visual search asymmetry (*60*).

In conclusion, our study sheds light on the complex relationship between visual feature coding and attention coding in the primate brain. Units exhibiting visual feature coding display a stronger attentional modulation of responses and interactions between brain areas compared to units not exhibiting feature coding, indicating a nuanced interplay between these cognitive processes. Future research exploring how these findings translate to behavioral outcomes and cognitive functions in both nonhuman primates and humans could offer valuable implications for understanding attentional processes and cognitive control in diverse contexts.

## MATERIALS AND METHODS

### Subjects

Two male rhesus macaques, weighing 12 and 15 kg, were used in the study. The monkeys were implanted under aseptic conditions with a post to fix the head and recording chambers over areas V4, IT cortex (including both TEO and TE), LPFC, and OFC. The localization of the chambers was based on magnetic resonance imaging (MRI) scans obtained before surgery. All experiments were performed with the approval of the Institutional Animal Care and Use Committee of Shenzhen Institutes of Advanced Technology, Chinese Academy of Sciences (no. SIAT-IRB-160223-NS-ZHH-A0187-003). This dataset has been analyzed in previous studies (*33*, *34*, *42*).

### Tasks and stimuli

Monkeys were trained to perform a free-gaze visual search task. A central fixation was presented for 400 ms, followed by a cue lasting 500 to 1300 ms. After a delay of 500 ms, the search array was on. The search array contained 11 items, including two targets, randomly selected from a total of 20 predefined locations. Monkeys were required to find either one of the two targets within 4000 ms and maintain fixation on the target for 800 ms to receive a juice reward. No constraints were placed on their search behavior to allow animals to perform the search naturally. Before the onset of the search array, monkeys were required to maintain a central fixation. The two target stimuli belonged to the same category as the cue stimulus, although they were distinct images. We used four categories of stimuli—face, house, flower, and hand—each comprising 40 images. The cue stimulus was randomly selected from the house or face stimuli with equal probability. The remaining nine stimuli in the search array were drawn from the other three categories. Each stimulus subtended an area of approximately 2° × 2°, with the hue, saturation in the HSV color space, aspect ratio, and luminance of these images matched across categories. The 20 locations, covering the visual field of eccentricities from 5° to 11°, included 18 locations located symmetrically in the left and right visual field, with 9 on each side and 2 locations on the vertical middle line (Fig. 1B).

A visually guided saccade task was used to map the peripheral RFs of the recorded units. Following a 400-ms central fixation, a stimulus randomly appeared in 1 of the 20 peripheral locations, and monkeys were required to make a saccade to the stimulus within 500 ms and maintain fixation on it for 300 ms to receive a reward (we also included an additional central location where the monkeys only needed to fixate, without making a saccade). In this task, we used the same faces and houses from the visual search task as stimuli. We compared responses in a time window of 50 to 200 ms after stimulus onset (100 to 200 ms for the OFC) to the baseline (−150 to 0 ms relative to stimulus onset) using a two-tailed Wilcoxon rank-sum test to determine whether each unit had a significant response to the stimuli in a specific peripheral RF location. Behavioral experiments were conducted using the MonkeyLogic software (University of Chicago, IL), which presented the stimuli, monitored the eye movements, and triggered the delivery of the reward.

### Electrophysiology

Single- and multi-unit spikes were recorded from V4, IT, LPFC, and OFC using 24- or 32-contact electrodes (V-Probe or S-Probe, Plexon Inc., Dallas, USA) in a 128-channel Cerebus system (Blackrock Microsystems, Salt Lake City, UT, USA). In most sessions, we recorded activities in two of the areas simultaneously. Neural signals were filtered between 250 and 5 kHz, amplified, and digitized at 30 kHz to obtain spike data. The recording locations in V4, IT, LPFC, and OFC were verified with MRI. Eye movements were recorded using an infrared eye-tracking system (iViewX Hi-Speed, SensoMotoric Instruments, Teltow, Germany) with a sampling rate of 500 Hz.

Neural data were sorted offline using the Offline Sorter software (Plexon Inc., Dallas, USA). Using its manual user interface, we semiautomatically evaluated PC analyses and individual waveform shapes based on the algorithms implemented in the software. Specifically, raw electrophysiological data were high-pass filtered (250 Hz) to isolate spike signals from low-frequency LFPs and noise. Spikes were detected using a threshold-based method, where thresholds were manually adjusted for each channel to exclude background noise while capturing all potential spikes. Detected waveforms were then characterized using features such as principal components analysis (PCA), energy, and peak-to-peak amplitude, with PCA primarily used to reduce dimensionality while preserving waveform variability critical for classification. Clustering of waveforms into putative single units was performed using the software’s built-in tools, which included automated methods such as *k*-means or Gaussian mixture models, as well as manual adjustments to refine boundaries. Clusters were validated by assessing inter-spike interval histograms, signal-to-noise ratios, and spike waveform consistency to confirm proper isolation of individual units. In addition, large-amplitude artifacts and overlapping spikes were excluded to ensure clean and reliable data. Sorted spike trains were subsequently exported as timestamps or continuous waveforms for downstream analyses. Post hoc quality control, including calculations of signal-to-noise ratio (*43*, *61*–*63*) and isolation distance (*64*, *65*), was conducted to ensure the robustness of spike sorting.

### Data analysis: Spike rate

Measurements of neural activity were obtained from spike density functions, which were generated by convolving the time of action potentials with a function that projects activity forward in time (growth = 1 ms, decay = 20 ms) and approximates an excitatory postsynaptic potential (*66*). The spike rate of each unit was normalized to its maximum spike rate across conditions.

### Data analysis: RF

The visual response to the cue and the search array in the free-gaze visual search task was assessed by comparing the firing rate during the post-stimulus period (50 to 200 ms after cue/array onset) to the corresponding baseline (−150 to 0 ms relative to cue/array onset) using a Wilcoxon rank-sum test. On the basis of these responses, we classified units into three categories of RFs:

1) Units with a focal foveal RF: These units responded solely to the cue in the foveal region (*P* < 0.05) but not to the search array that included items in the periphery (*P* > 0.05).

2) Units with a broad foveal RF: These units responded to both the cue and the search array.

3) Units with a peripheral RF: These units only responded to the search array (*P* < 0.05) but not to the cue (*P* > 0.05). The RFs of these units were additionally mapped on the basis of their activities in the visually guided saccade task (see above).

Units not classified into one of the categories were excluded from further analysis. In this study, our focus was on units with a focal foveal RF (category 1) because we aimed to analyze the visual feature coding properties and compare fixations on targets versus distractors. We also analyzed the response in the V4 peripheral RF because V4 (but not TEO or TE) had a focal peripheral RF (i.e., only one search item was encompassed by the RF).

We used the visual search task (rather than the saccade task) to map the foveal RFs, and the monkeys’ cognitive state may differ between the cue and search array presentations. Consequently, responses to the cue and search array may not be directly comparable for sensory mapping. However, we observed consistent visual feature coding results during both the cue and search phases (Fig. 2, F to K). We also confirmed that the response in the foveal RF was consistent between the visual search and saccade tasks.

### Data analysis: Category selectivity

We determined the category selectivity of each unit by comparing the response to face cues versus house cues in a time window of 50 to 200 ms after cue onset (Wilcoxon rank-sum test, *P* < 0.05). We further imposed a second criterion using an SI similar to indices used in previous IT studies (*67*, *68*). For each unit with a foveal RF, the response to face stimuli (*R*_face_) or house stimuli (*R*_house_) was calculated using the visual search task by subtracting the mean baseline activity (−150 to 0 ms relative to the onset of the cue) from the mean response to the face or house cue (50 to 200 ms after the onset of the cue). For each unit with a peripheral RF, *R*_face_ and *R*_house_ were calculated using the visually guided saccade task by subtracting the mean baseline activity (−150 to 0 ms relative to the peripheral stimulus onset) from the mean response to the saccade target (50 to 200 ms after the onset of the saccade target). The SI was then defined as (*R*_face_ − *R*_house_)/(*R*_face_ + *R*_house_). SI was set to 1 when *R*_face_ > 0 and *R*_house_ < 0 and to −1 when *R*_face_ < 0 and *R*_house_ > 0. Face-selective units were required to have an *R*_face_ at least 130% of *R*_house_ (i.e., the corresponding SI was greater than 0.13). Similarly, house-selective units were required to have an *R*_house_ at least 130% of *R*_face_ (i.e., the corresponding SI was smaller than −0.13). Units were labeled as non–category selective if the response to face cues versus house cues was not significantly different (*P* > 0.05). The remaining units that did not fit into any of the aforementioned types were classified as undefined units (i.e., there was a significant difference but did not meet the second criterion).

### Data analysis: Selection of axis-coding units

We used the well-known DNN implementation based on the VGG-16 convolutional neural network architecture (*69*) to extract features for each image [replicated by AlexNet (*70*) and ResNet (*71*); fig. S5]. Following the same procedure (*72*), fine-tuning of the top layer of each DNN was performed to confirm that the pretrained model was able to discriminate the stimuli and ensure that the pretrained model was suitable as a feature extractor. We also used the fine-tuning accuracy to determine the most suitable model for feature extraction. There were 512 free parameters (i.e., DNN features) for VGG-16, 256 for AlexNet, and 2048 for ResNet (note that we averaged over the width and height, retaining only the depth).

To identify axis-coding units (i.e., units that encode a linear combination of visual features), we used PLS regression with DNN feature maps. We used the mean firing rate in a time window of 50 to 150 ms after fixation onset as the response to each fixation (see also Fig. 2, A and C, for reference) (*37*, *38*). The PLS method has been shown to be effective to study the neural response to DNN features (*25*, *36*). We used four components for each layer, explaining at least 60% of variance. We used a permutation test with 1000 runs to determine whether a unit encoded a significant axis–coding model (i.e., the unit encoded the dimensions of the feature space, demonstrating axis coding). In each run, we randomly shuffled the stimulus labels and used 50% of the stimuli as the training dataset. We used the training dataset to construct a model (i.e., deriving regression coefficients), predicted responses using this model for each stimulus in the remaining 50% of stimuli (i.e., test dataset), and computed the Pearson correlation between the predicted and actual response in the test dataset. The distribution of correlation coefficients computed with shuffling (i.e., null distribution) was eventually compared to the one without shuffling (i.e., observed response). If the correlation coefficient of the observed response was greater than 95% of the correlation coefficients from the null distribution, then this axis-coding model was considered significant. The correlation coefficient could also indicate the strength of axis coding and thus be compared between different units.

### Data analysis: Selection of attention-selective units

We used the mean firing rate in a time window of 150 to 225 ms after fixation onset as the response to each fixation. For each unit, if there was a significant difference in response (determined using a two-tailed Wilcoxon signed-rank test, with a significance threshold of *P* < 0.05) between fixations on targets and fixations on distractors, then it was classified as an attention-selective unit. Similarly, for V4 units with a focal peripheral RF (as described above), we compared the response between targets and distractors within the RF in the same time window as for foveal units. Last, we calculated the attentional effect as the difference in firing rate between the same stimuli when they served as targets versus distractors.

### Data analysis: RSA

For the RSA (*39*), DMs are symmetrical matrices representing dissimilarity between all pairs of stimuli. In a DM, larger values indicate greater dissimilarity between pairs, with the smallest value possible indicating similarity of a condition to itself (dissimilarity of 0). We used Pearson correlation to calculate DMs and Spearman’s correlation to calculate the correspondence between DMs, as Spearman’s correlation does not assume a linear relationship (*73*). Specifically, the dissimilarity value (1 − Pearson’s *r*) for the neural DM was calculated using the mean response of all axis-coding units within the same time window as visual feature coding, between each pair of stimuli (Fig. 2L). The dissimilarity value (1 − Pearson’s *r*) for the DNN DM was calculated using all features (i.e., unit activations) from the DNN layer Pool5. To assess the significance of the correspondence between the neural DMs (Fig. 2M) and between the neural and DNN DMs (Fig. 2N), we used permutation tests with 1000 runs. In each run, stimulus labels were randomly shuffled, and the correlation between DMs was recalculated. The distribution of correlation coefficients generated from shuffling (i.e., the null distribution; shaded in gray in Fig. 2, M and N) was then compared to the observed correlation coefficient (i.e., unshuffled; connected dots in Fig. 2, M and N). If the observed correlation coefficient exceeded 95% of the coefficients from the null distribution, then it was deemed significant. Bonferroni correction was applied to account for multiple comparisons.

### Data analysis: Representational distance

We used the mean firing rate in a time window of 50 to 150 ms after fixation onset as the response to each fixation. For a population of units, we calculated the Euclidean distance between units. Change in population geometry was described using the cosine angle between the neuronal vectors: $\text{cosθ}=\frac{a\cdot b}{|a\|b|}$, where *a* and *b* are the neuronal vectors for different conditions.

### Data analysis: Spike-LFP coherence

We implemented the spike-LFP coherence analysis using the Chronux toolbox (www.chronux.org) in MATLAB. We used a single Hanning taper across frequencies, but we derived similar results using multitaper methods for higher frequencies (>25 Hz) (*74*). Coherence between two signals, *x* and *y*, was calculated using the following formula

$$C_{\mathrm{xy}}\left(f\right)=\frac{S_{\mathrm{xy}}\left(f\right)}{\sqrt{S_{x}\left(f\right)\cdot S_{y}\left(f\right)}}$$

where *S_x_*(*f*) and *S_y_*(*f*) denote the autospectra and *S_xy_*(*f*) represents the cross-spectrum of the two signals *x* and *y*. Autospectra and cross-spectra were averaged across fixations before the coherence calculation. We used a 200-ms time window for each fixation (0 to 200 ms relative to fixation onset). Notably, we used an equal number of fixations and an equal number of spikes between conditions to calculate the coherence for a given pair of recording sites, thus eliminating bias from different sample sizes. To avoid spikes contributing to the LFP recorded on the same electrode, we used signals from two different electrodes to calculate the coherence. Furthermore, we did not select LFPs based on their category selectivity, attention selectivity, or the selectivity of the associated units (e.g., the LFP signals could come from contacts with both axis-coding and nonaxis-coding units).

### Data analysis: Granger causality

We used the open-source MATLAB toolbox “Granger causal connectivity analysis” (*75*) for our study. Frequency-domain Granger causalities were calculated during the same period as in coherence analysis between spikes and LFP across various brain areas. Two preprocessing steps, namely, detrending and demeaning, were applied to the LFPs. The Granger causal connectivity analysis toolbox functions “cca_detrend” and “cca_rm_ensemblemean” were used to subtract the best-fitting line of the LFP for each fixation and the ensemble mean of the LFP, respectively. Subsequently, we used a KPSS test to assess the stationarity of the LFP data after preprocessing, and any nonstationary LFP data were excluded from the analysis. Frequency-domain Granger causality was calculated on the basis of the stationary LFPs after preprocessing using the function “cca_pwcausal” from the toolbox. The calculation is as follows

$$G_{j\to i}\left(f\right)=-\text{ln}\left\{1-\frac{\left[\Sigma_{jj}-\left(\frac{\Sigma_{ij}^{2}}{\Sigma ii}\right)\right]{|H_{ij}\left(f\right)|}^{2}}{S_{ii}\left(f\right)}\right\}$$

where *S_ii_*(*f*) is the power spectrum of variable *i* at frequency *f*, *H* is the transfer matrix, and Σ is the noise covariance matrix (*75*).
