# Supplementary material for: Distinct attentional characteristics of neurons with visual feature coding in the primate brain
Source: Sci Adv. 2025 Mar 21;11(12):eadq0332. doi: 10.1126/sciadv.adq0332 (PMC11927616; doi:10.1126/sciadv.adq0332)
Supplement: Supplementary file 1 — Figs. S1 to S14 [file sciadv.adq0332_sm.pdf]

Science Advances
AAAS

Supplementary Materials for

Distinct attentional characteristics of neurons with visual feature coding in the primate brain

Jie Zhang *et al.*

Corresponding author: Jie Zhang, zjie@wustl.edu; Huihui Zhou, zhouhh@pcl.ac.cn; Shuo Wang, shuowang@wustl.edu

Sci. Adv. **11**, eadq0332 (2025)
DOI: 10.1126/sciadv.adq0332

This PDF file includes:

Figs. S1 to S14

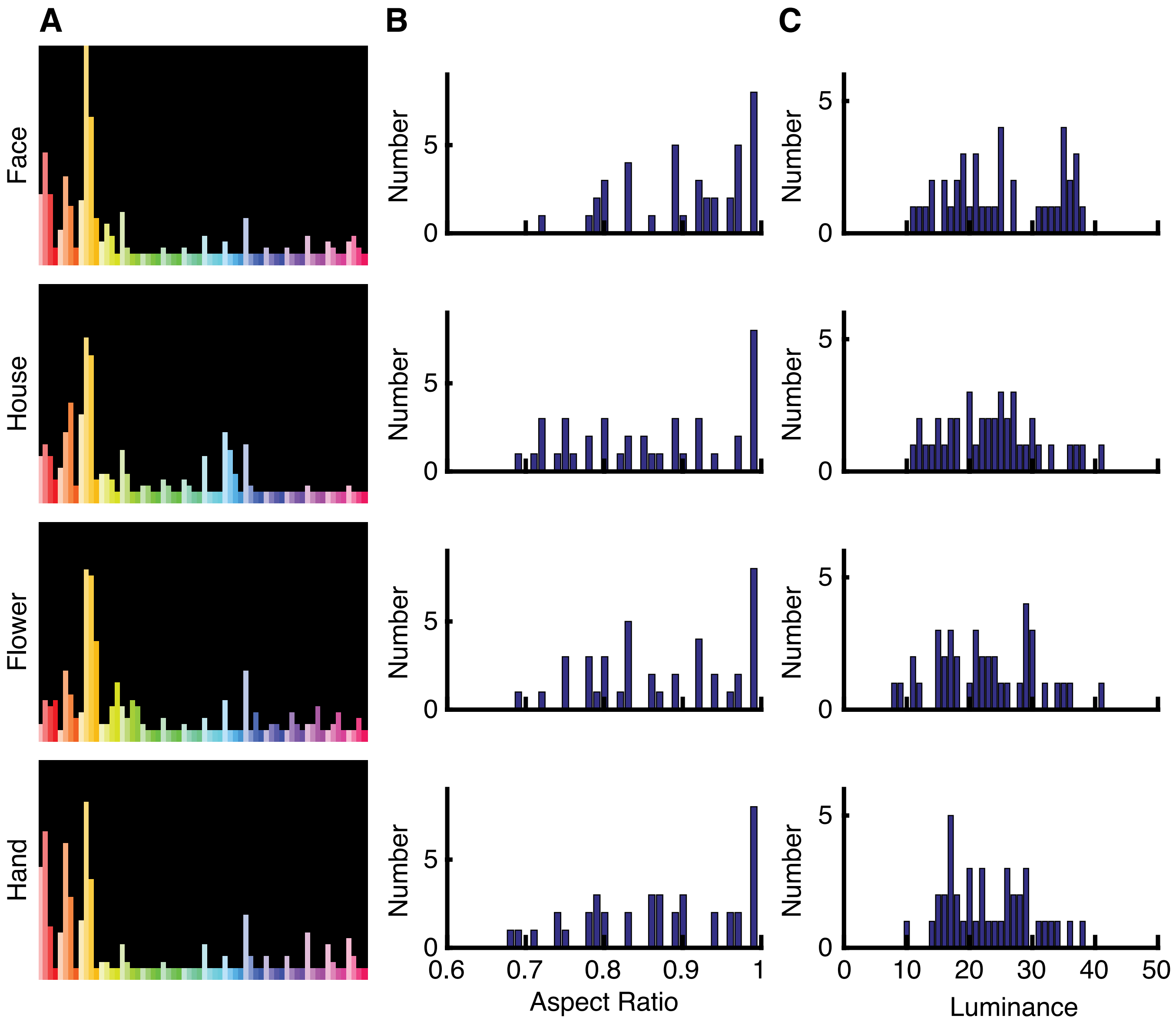

fig. S1. Characterization of the stimuli. (A) The stimuli from different categories did not exhibit significant differences in pixel-wise hue and saturation (χ^2-test: $P > 0.05$). **(B)** The stimuli from different categories did not exhibit significant differences in aspect ratio ($P > 0.05$). **(C)** The stimuli from different categories did not exhibit significant differences in luminance ($P > 0.05$). Shown are histograms for each stimulus category.

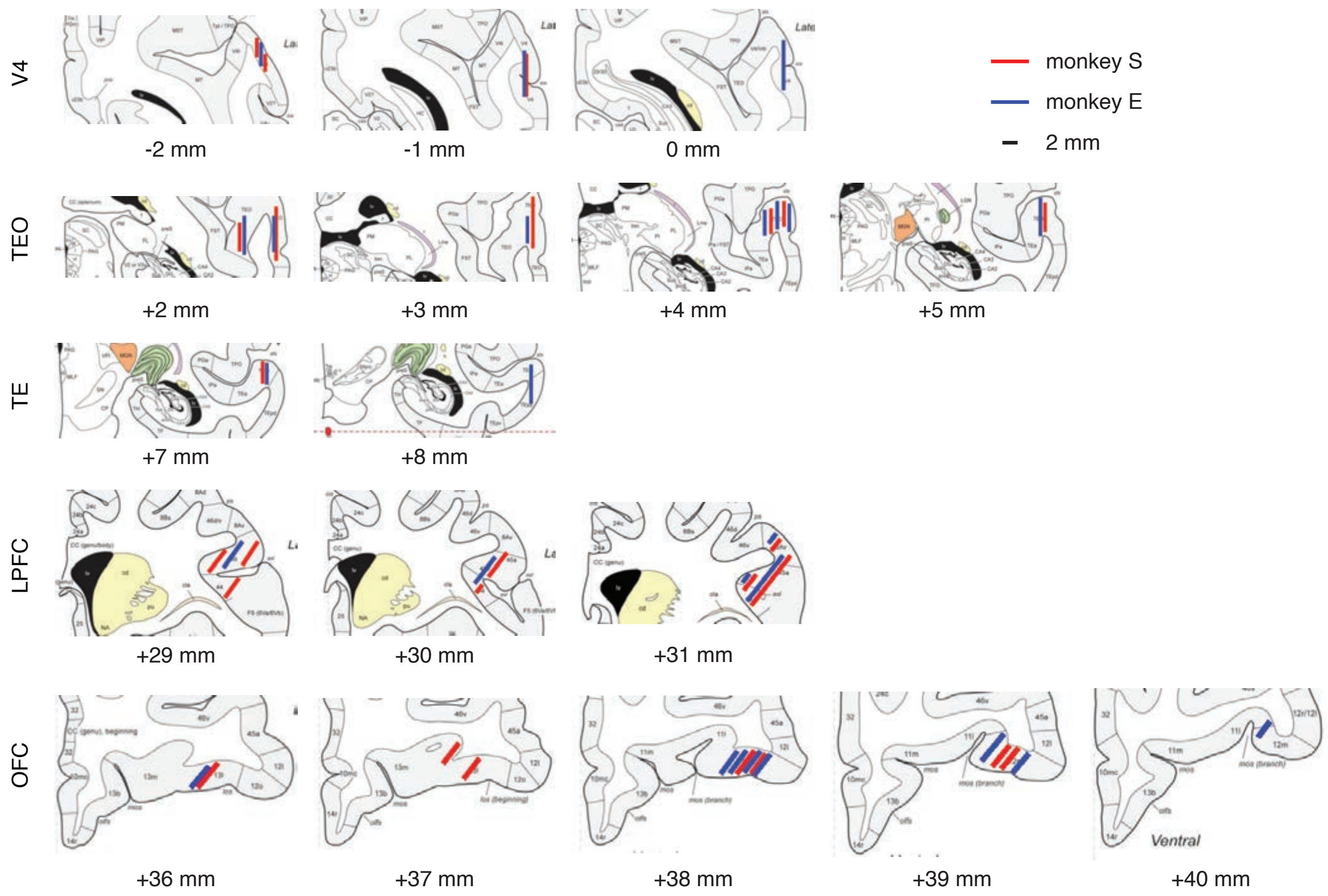

fig. S2. Recording sites in two monkeys overlaid on the atlas of the rhesus monkey brain in stereotaxic coordinates. The red and blue lines represent the estimated spatial range of recordings in monkey S and monkey E, respectively. The numbers below indicate the rostral (+) or caudal (-) distances of the slices from the infraorbital ridge (Ear Bar Zero).

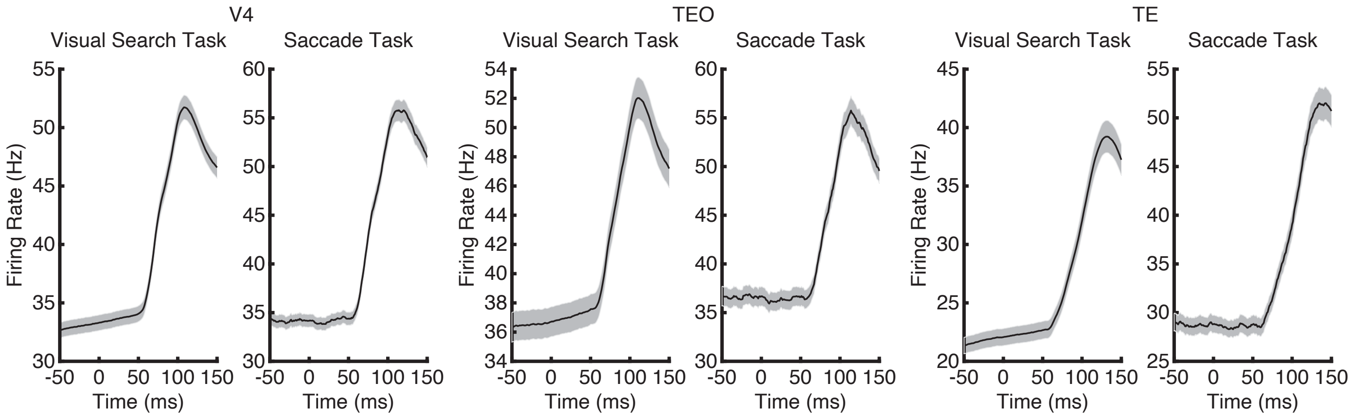

fig. S3. Response in the visual search versus saccade tasks. The units with a focal foveal receptive field (RF) were selected using the cue from the visual search task. The units with a focal foveal RF selected using the cue from the visual search task (i.e., the units used in the present study) exhibited a similar response in the saccade task. Furthermore, units with a focal foveal RF selected from the saccade task could largely be selected from the visual search task. Specifically, 95.63% of units from V4, 98.17% of units from TEO, and 99.79% of units selected from the saccade task could also be identified in the visual search task. Therefore, the units classified as having a focal foveal RF were mostly the same regardless of the method used.

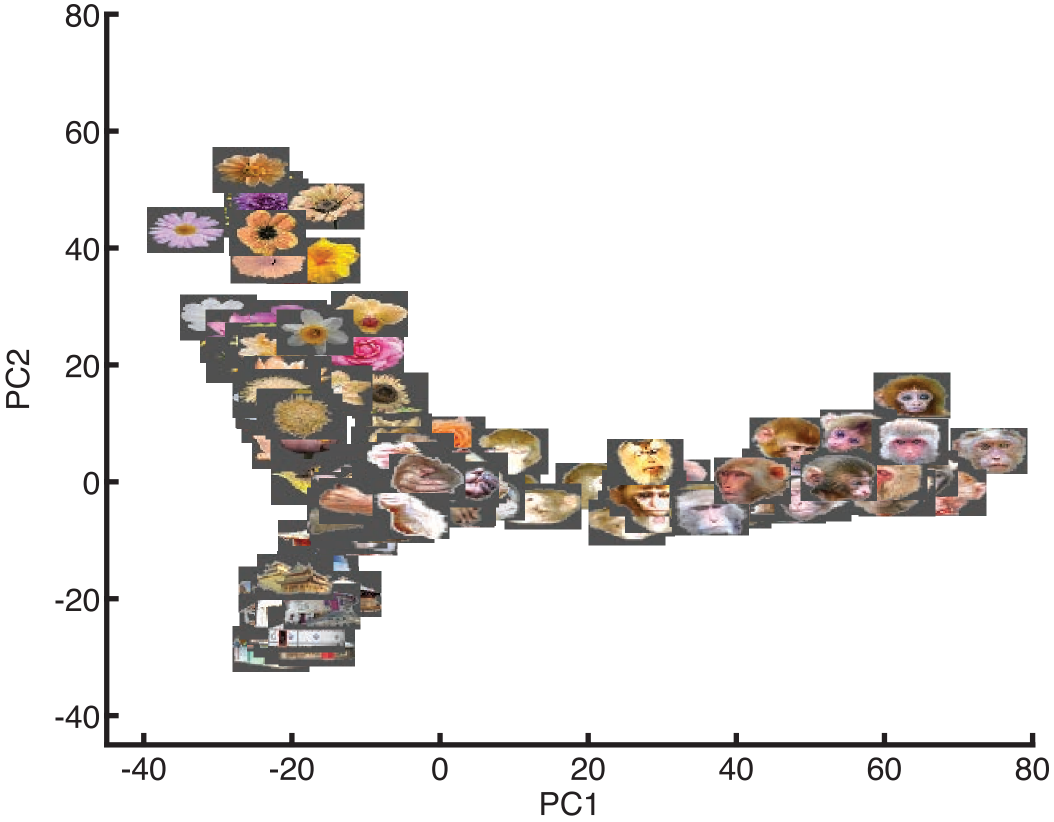

fig. S4. Distribution of stimuli in the principal component analysis (PCA) feature space. As shown, the first principal component (PC1) represents changes from inanimate (flowers, houses) to animate (faces) stimuli.

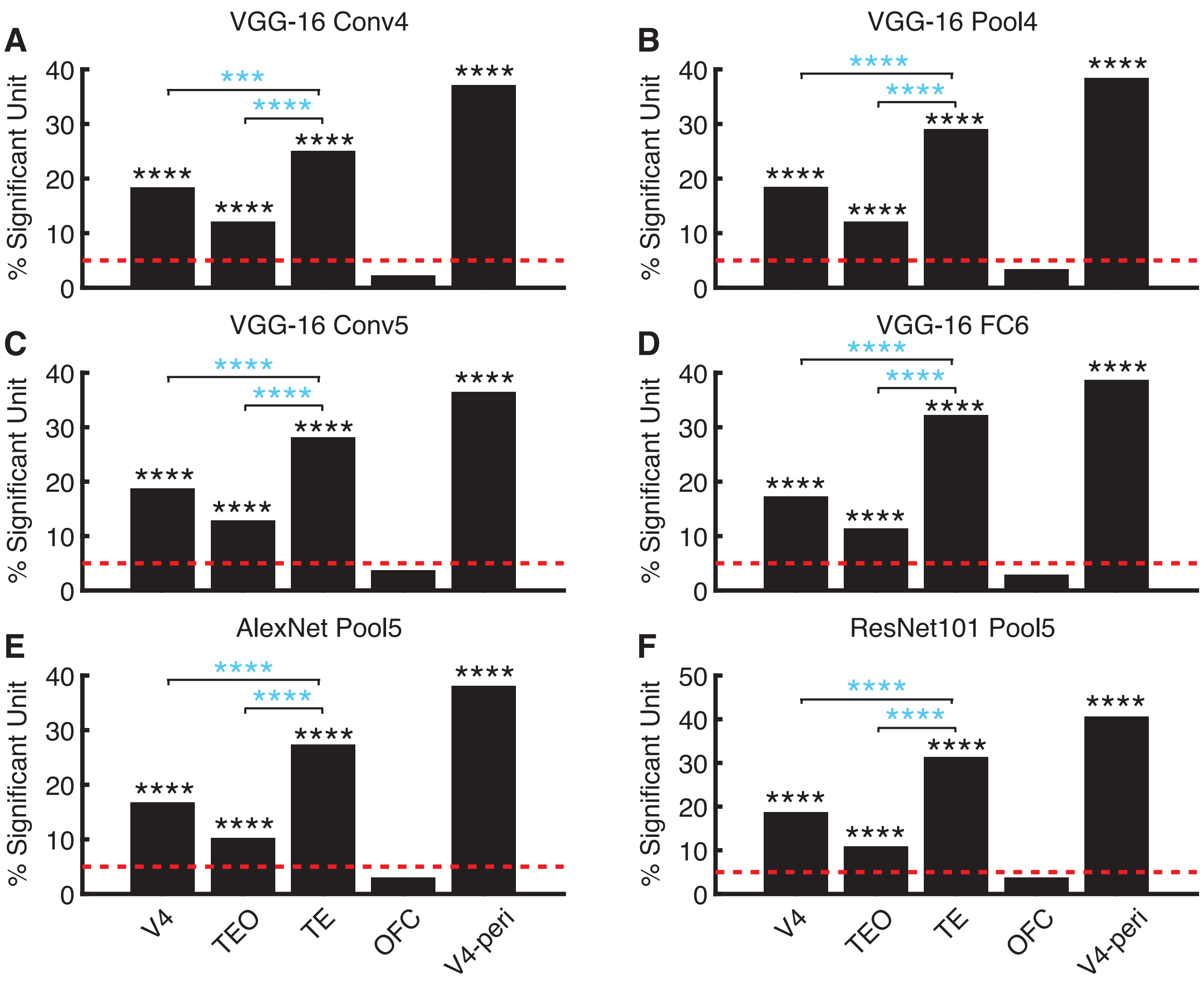

fig. S5. Control analysis for axis coding. (A-D) The proportion of axis-coding units for each brain area using features from different VGG-16 layers. **(E)** The proportion of axis-coding units for each brain area using features from the AlexNet. **(F)** The proportion of axis-coding units for each brain area using features from the ResNet. Black asterisks indicate a significant above-chance (5%) number of units (binomial test). Blue asterisks indicate a significant difference between brain areas (χ^2-test). ****: P < 0.0001.

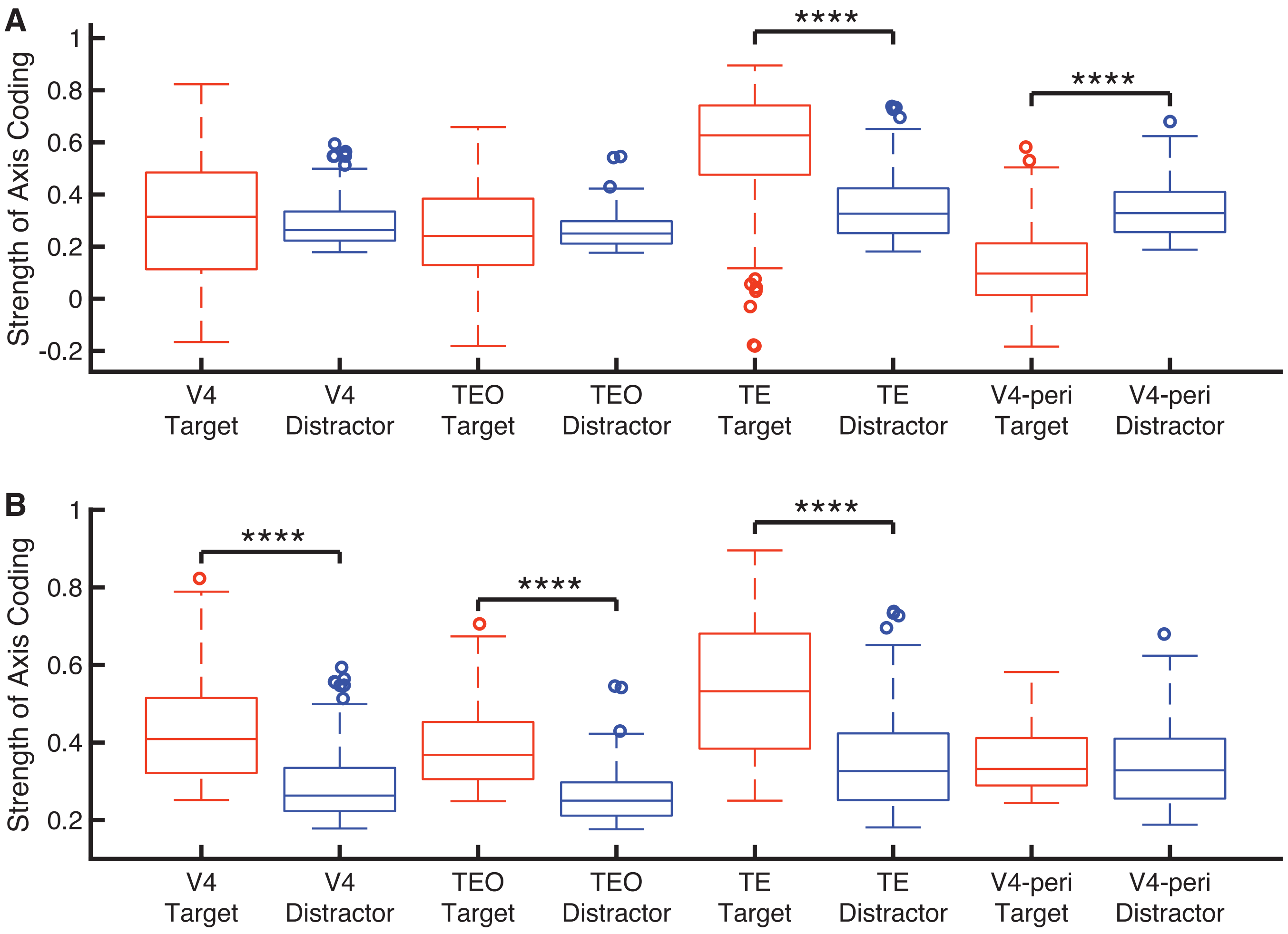

fig. S6. Strength of axis coding. **(A)** For both targets and distractors, axis-coding units were selected based on fixations on distractors (replotted and reorganized from **Fig. 2F, G**). We found that the strength of axis coding was significantly higher for fixations on targets in TE, suggesting that attention increases feature coding strength. However, we did not observe a significant difference in V4 or TEO, likely because the axis-coding units were selected based on fixations on distractors (the strength of axis coding for fixations on targets represented a more out-of-sample prediction and was therefore weaker). **(B)** Axis-coding units were selected separately using fixations on targets and fixations on distractors. As a control, we also compared the strength of axis coding using axis-coding units selected separately based on fixations on distractors and targets. Indeed, we found that the strength of axis coding was greater for targets than for distractors, consistent with the notion that attention enhances feature coding strength. Notably, the pattern of results (i.e., the relative strength of axis coding across brain areas) was similar regardless of whether axis-coding units were selected based on fixations on targets or distractors. Furthermore, we observed a lower strength of axis coding for V4 peripheral units compared to V4, TEO,

and TE foveal units when axis-coding units were selected based on fixations on targets, confirming that V4 peripheral units were suppressed during fixations on targets. Legend conventions as in **Fig. 2F-K**.

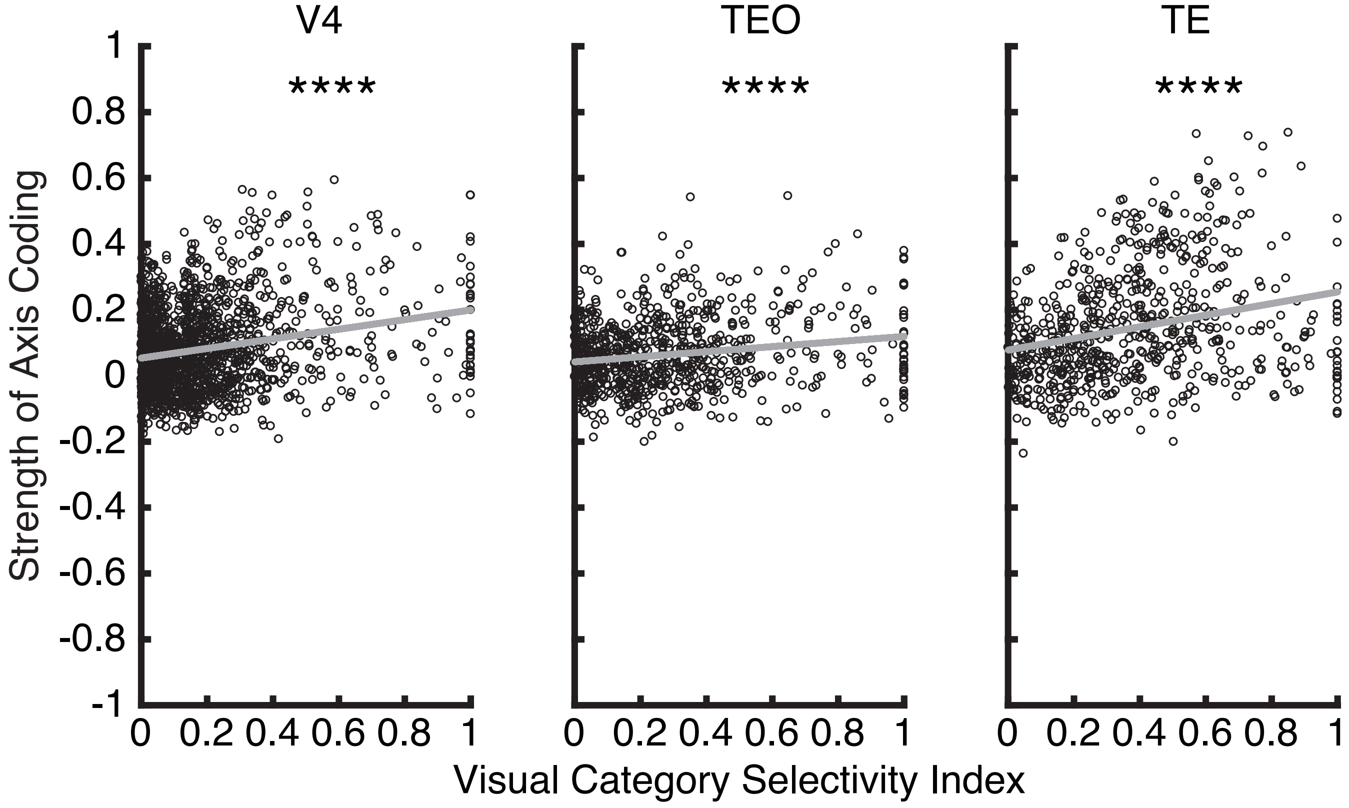

fig. S7. Correlation between the strength of axis coding and the strength of visual category selectivity. Each dot represents a unit. Asterisks indicate a significant Pearson correlation. ****: $P < 0.0001$. All units: V4: $r = 0.234$, $P = 1.45 \times 10^{-21}$; TEO: $r = 0.182$, $P = 4.07 \times 10^{-7}$; TE: $r = 0.261$, $P = 1.15 \times 10^{-11}$. Axis-coding units: V4: $r = 0.352$, $P = 5.33 \times 10^{-10}$; TEO: $r = 0.211$, $P = 0.045$; TE: $r = 0.357$, $P = 1.10 \times 10^{-7}$.

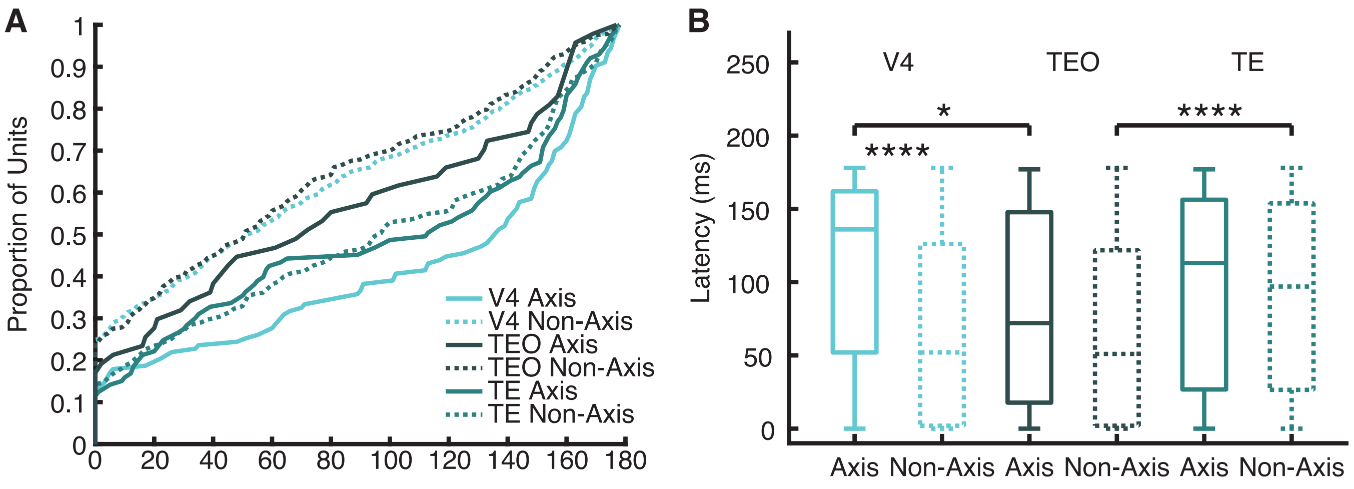

fig. S8. Differential latency. For each unit, the latency was defined as the first 20-ms bin out of twelve successive bins that had a significantly different response between targets versus distractors using a one-tailed Wilcoxon signed-rank test with a significance threshold of $P < 0.05$. **(A)** Cumulative distribution of differential latencies. **(B)** Group summary. Asterisks indicate a significant difference between groups using a two-tailed two-sample *t*-test. *: $P < 0.05$, and ****: $P < 0.0001$. The enhanced neural response to targets occurred significantly earlier in TEO axis-coding units compared to V4 axis-coding units, and it also occurred significantly earlier in TEO non-axis-coding units compared to TE non-axis-coding units. Additionally, the enhanced neural response to targets was significantly earlier in V4 non-axis-coding units compared to V4 axis-coding units.

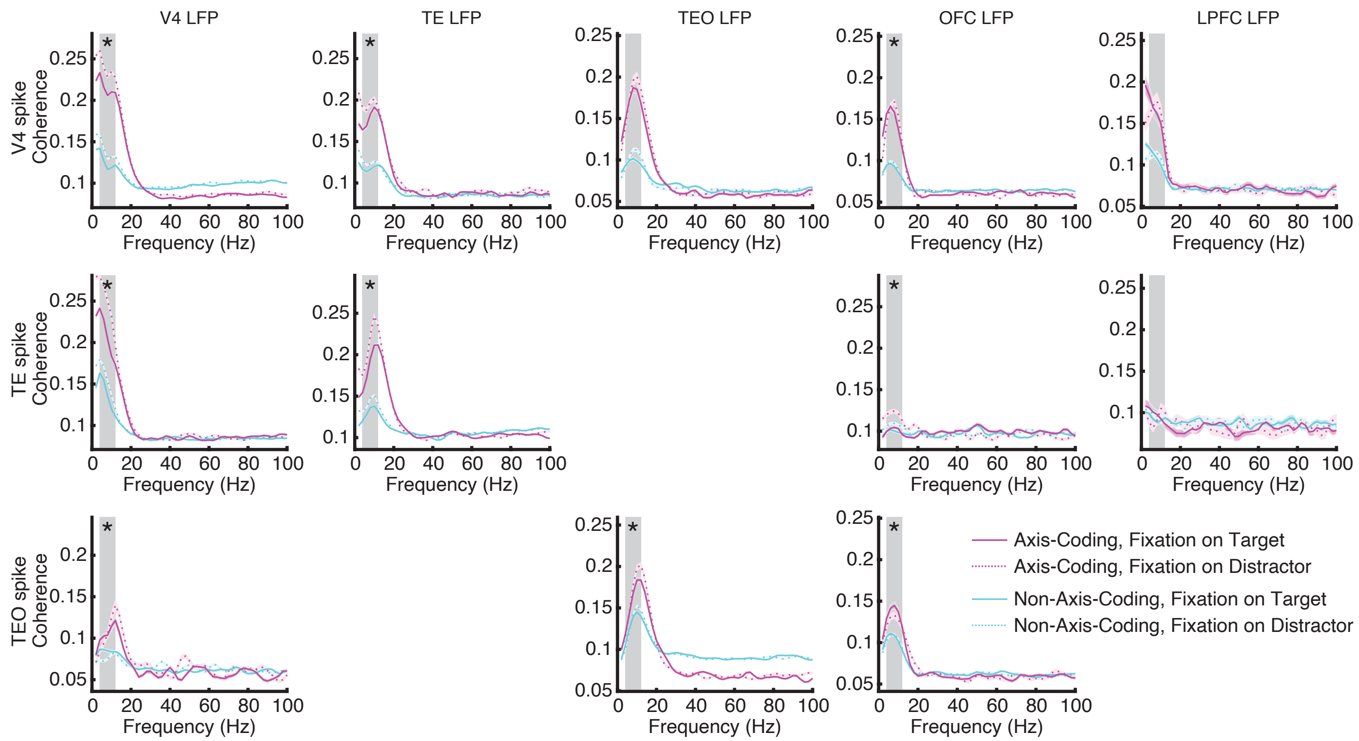

fig. S9. Spike-LFP coherence separately for TE and TEO. Legend conventions as in **Fig. 6**.

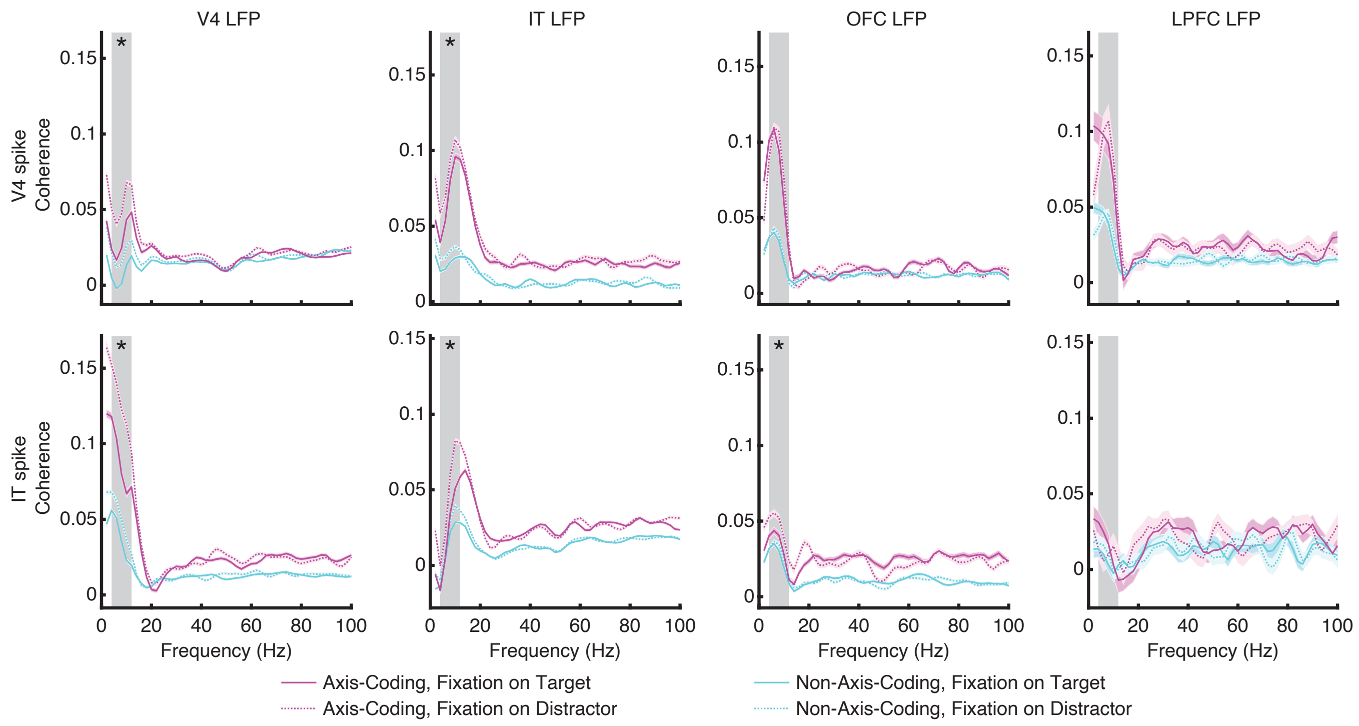

fig. S10. Control analysis for spike-LFP coherence with baseline subtraction. Spikes desynchronized with LFPs in the theta frequency band for fixations on targets compared to fixations on distractors. Axis-coding units demonstrated a stronger target-induced desynchronization ($Coherence_{Distractor} - Coherence_{Target}$) between V4 spike and V4 LFP (two-tailed two-sample *t*-test: $t(29404) = 12.84$, $P = 1.19\times10^{-37}$), between V4 spike and IT LFP ($t(7801) = 3.0$, $P = 6.52\times10^{-5}$), between IT spike and V4 LFP ($t(7979) = 17.08$, $P = 3.01\times10^{-64}$), between IT spike and IT LFP ($t(22658) = 15.74$, $P = 1.63\times10^{-55}$), and between IT spike and OFC LFP ($t(5037) = 4.6$, $P = 4.4\times10^{-6}$). All fixations and spikes were included in this analysis; however, we obtained qualitatively the same results when using an equal number of fixations and spikes across conditions. Legend conventions as in **Fig. 6**.

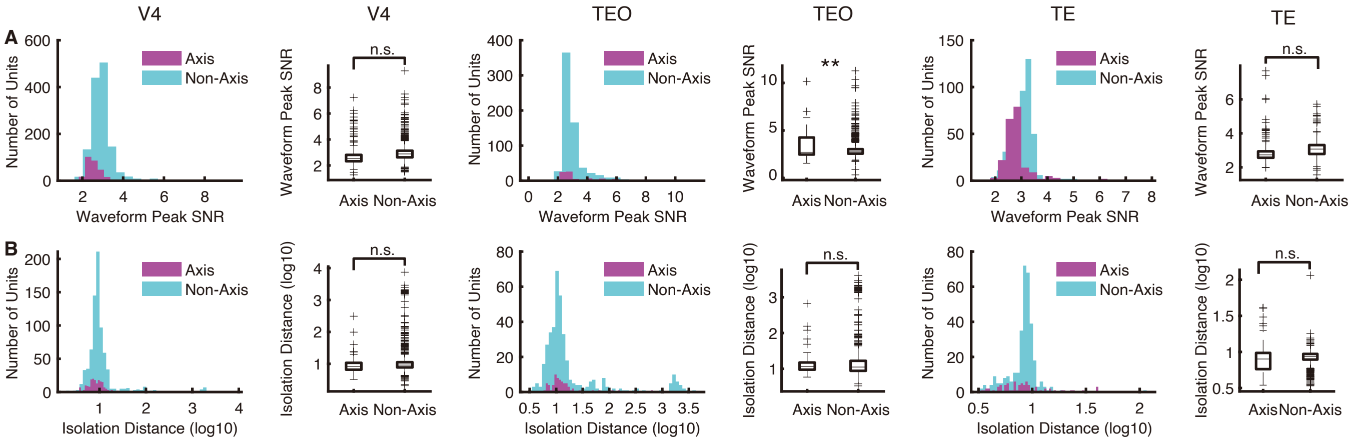

fig. S11. Spike sorting quality for axis-coding and non-axis-coding units. (A) Signal-to-noise ratio (SNR) of the sorted units (43, 61-63). The peak of the mean spike waveform was considered the signal. Since spikes were stored separately from local field potentials (LFPs, which were low-pass filtered) in our recordings, we used the two ends of the spike waveform (0.17 to 0.3 ms before the spike detection threshold and 0.5 to 1.27 ms after the spike detection threshold) as substitutes for high-pass filtered raw signals to estimate the noise. **(B)** Isolation distance. Isolation distance was calculated based on (64, 65). If a cluster contains n_C cluster spikes, the isolation distance of the cluster is the D^2 value of the n_C th closest noise spike. Isolation distance is therefore the radius of the smallest ellipsoid from the cluster center containing all of the cluster spikes and an equal number of noise spikes. As such, isolation distance estimates how distant the cluster spikes are from the other spikes recorded on the same electrode. Isolation distance is not defined for cases in which the number of cluster spikes is greater than the number of noise spikes. The left panel shows the distribution of the values. On each box in the right panel, the central mark is the median across units, the edges of the box are the 25th and 75th percentiles, the whiskers extend to the most extreme data points the algorithm considers to be not outliers, and the circles denote the outliers. Asterisks indicate a significant difference between brain axis-coding and non-axis-coding units using a one-tailed (axis-coding > non-axis-coding) two-sample *t*-test. **: P < 0.01 and ****: P < 0.0001. n.s.: not significant.

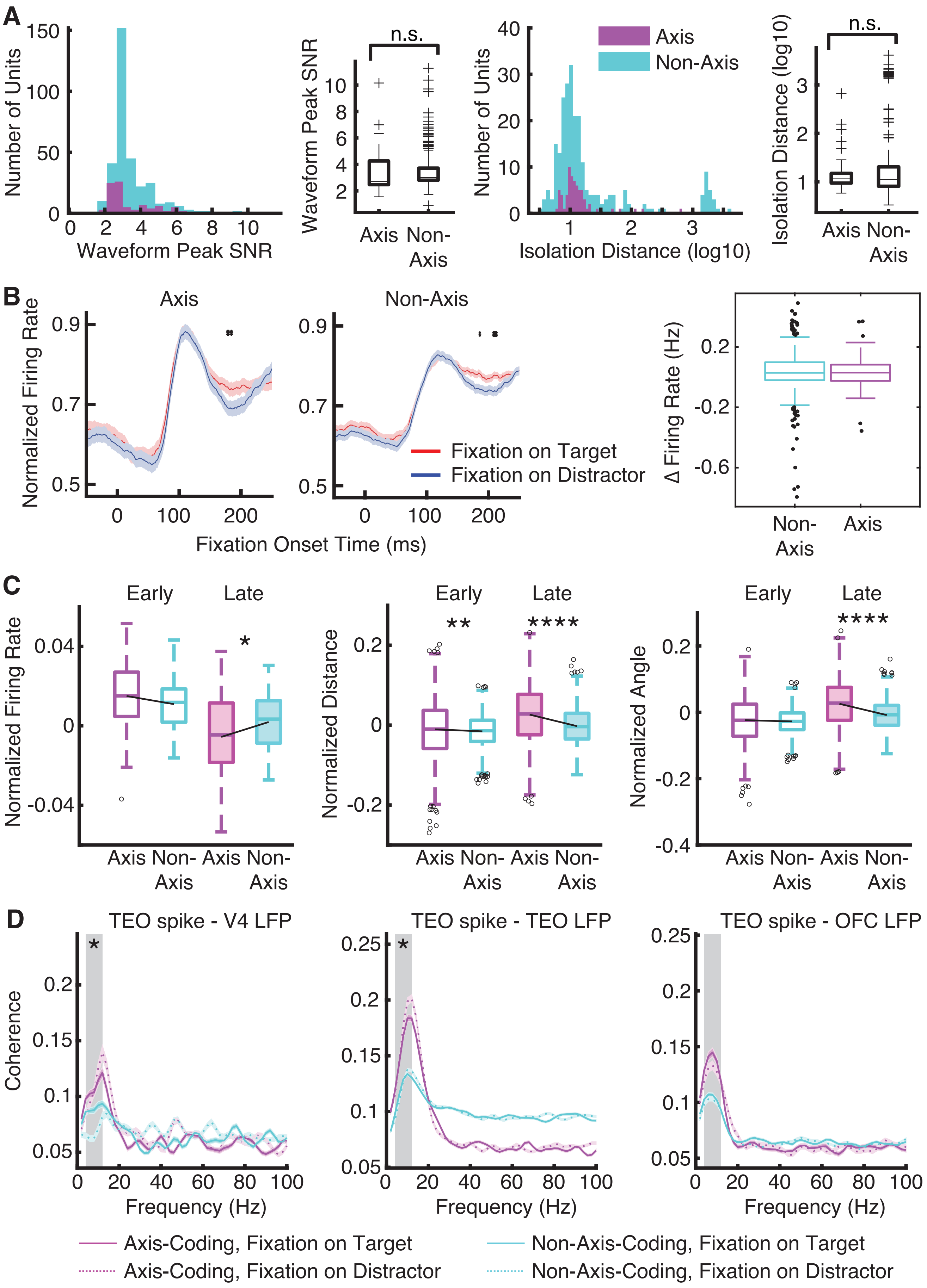
A
Number of Units
Waveform Peak SNR
n.s.
Axis
Non-Axis
Isolation Distance (log10)
B
Axis
Non-Axis
Normalized Firing Rate
Fixation Onset Time (ms)
Fixation on Target
Fixation on Distractor
Δ Firing Rate (Hz)
C
Early
Late
Normalized Firing Rate
Normalized Distance
Normalized Angle
D
TEO spike - V4 LFP
TEO spike - TEO LFP
TEO spike - OFC LFP
Coherence
Frequency (Hz)
Axis-Coding, Fixation on Target
Axis-Coding, Fixation on Distractor
Non-Axis-Coding, Fixation on Target
Non-Axis-Coding, Fixation on Distractor

fig. S12. Control results for TEO with matched spike sorting properties. (A) Signal-to-noise ratio (SNR) and isolation distance of the sorted units. Legend conventions as in **fig. S11**. Here, we selected the top 50% of non-axis-coding units with the highest SNR to match the axis-coding units. **(B)** Attentional effect. Legend conventions as in **Fig. 3**. **(C)** Differential attentional modulation of neuronal representational geometry. Legend conventions as in **Fig. 5**. **(D)** Spike-LFP coherence. Legend conventions as in **Fig. 6**.

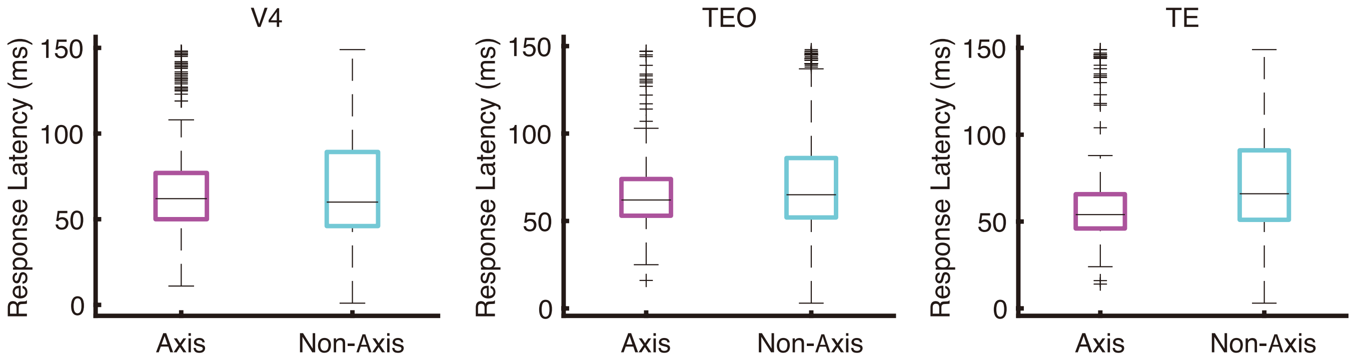

fig. S13. Response latency (34) of visual responsiveness between axis-coding and non-axis-coding units. On each box, the central mark is the median across units, the edges of the box are the 25th and 75th percentiles, the whiskers extend to the most extreme data points the algorithm considers to be not outliers, and the circles denote the outliers. Across brain areas, no significant differences were observed between axis-coding and non-axis-coding units.

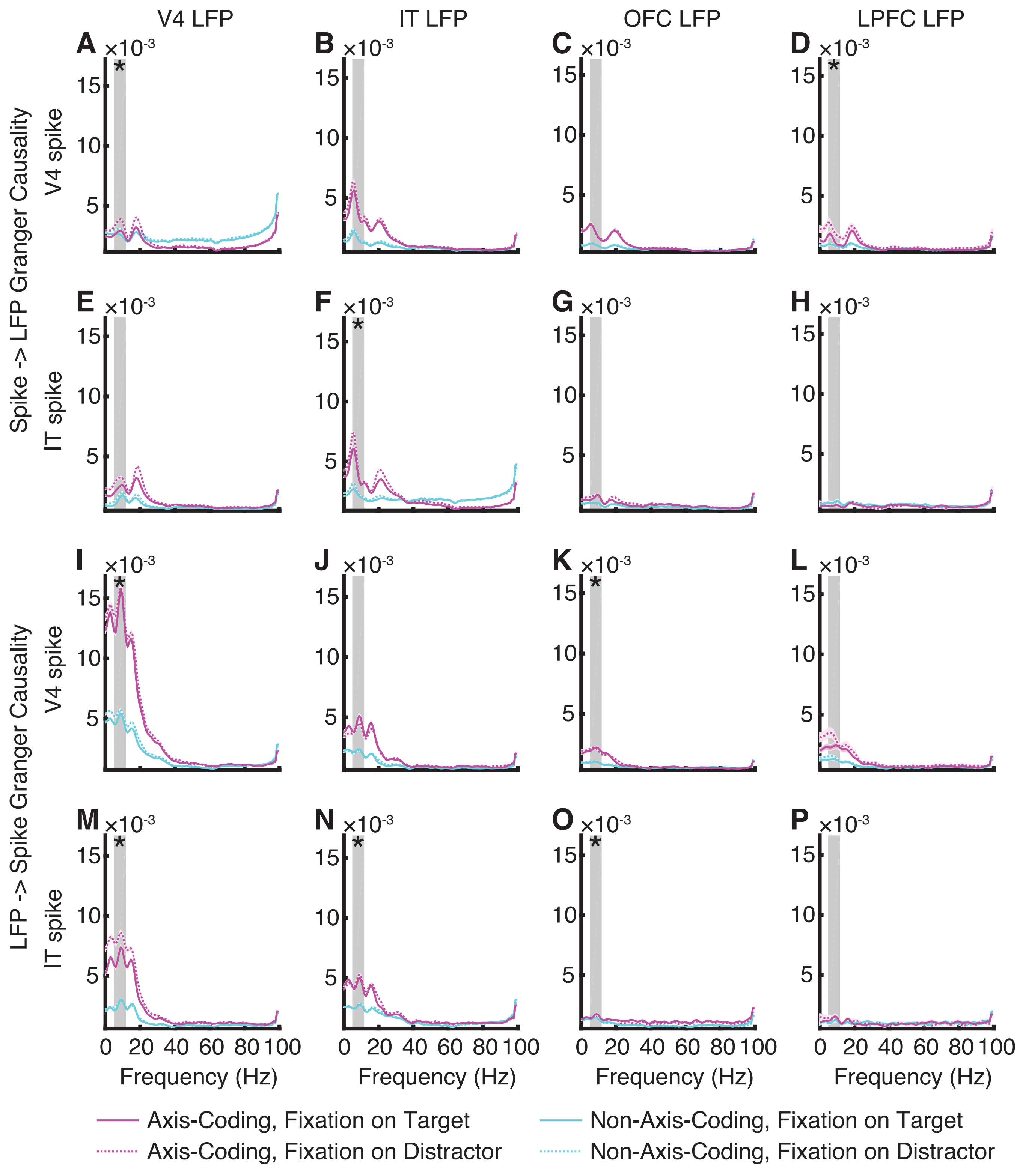

fig. S14. Granger causality. (A) V4 spike influence on V4 LFP. **(B)** V4 spike influence on IT LFP. **(C)** V4 spike influence on OFC LFP. **(D)** V4 spike influence on LPFC LFP. **(E)** IT spike influence on V4 LFP. **(F)** IT spike influence on IT LFP. **(G)** IT spike influence on OFC LFP. **(H)** IT spike influence on LPFC LFP. **(I)** V4 LFP influence on V4 spike. **(J)** IT LFP influence on V4 spike. **(K)** OFC LFP

influence on V4 spike. **(L)** LPFC LFP influence on V4 spike. **(M)** V4 LFP influence on IT spike. **(N)** IT LFP influence on IT spike. **(O)** OFC LFP influence on IT spike. **(P)** LPFC LFP influence on IT spike. Legend conventions as in **Fig. 6**.
